# REGγ Suppresses Ferroptosis and Induces Drug Resistance by Degrading WDR6 in Chondrosarcoma

**DOI:** 10.1002/advs.76293

**Published:** 2026-06-26

**Authors:** Fanrong Liu, Shihui Shen, Dongxia Li, Peilin Shi, Jiaming Xu, Tongxin Lv, Yingying Du, Jingwei Zhang, Bo Yang, Hansen Chen, Zhuoya Peng, Wenjie Ren, Lu Zhang, Sheng Cheng, Tongjin Yin, Wencai Sheng, Lei Li, Juan Wu, Qingcheng Yang, Shuogui Xu, Wei Zheng

**Affiliations:** ^1^ Department of Orthopedics Shanghai Sixth People's Hospital Affiliated to Shanghai JiaoTong University School of Medicine Shanghai China; ^2^ College of Medicine Southwest JiaoTong University Chengdu China; ^3^ East China Normal University‐Shanghai Putuo District Central Hospital Collaborative Research Center For Translational Medicine Shanghai Putuo District Central Hospital Shanghai China; ^4^ Zhejiang University‐University of Edinburgh Institute (ZJU‐UoE Institute) Zhejiang University Haining Zhejiang China; ^5^ Shanghai Key Laboratory of Regulatory Biology Institute of Biomedical Sciences School of Life Sciences East China Normal University Shanghai China; ^6^ College of Metrology Measurement and Instrument China Jiliang University Hangzhou Zhejiang China; ^7^ Department of Emergency and Trauma the First Affiliated Hospital of Naval Medical University Shanghai China; ^8^ Department of Pediatrics Affiliated Hospital 6 of Nantong University Pediatrics Department of Yancheng Third People's Hospital Yancheng China; ^9^ Department of Pharmacy The General Hospital of Western Theater Command Chengdu Chengdu China

**Keywords:** chondrosarcoma, combination therapy, drug resistance, ferroptosis, REGγ

## Abstract

Chondrosarcoma (CS) is a malignant bone tumor for which treatment efficacy remains clinically challenging owing to chemotherapy resistance. Ferroptosis, an iron‐dependent form of regulated cell death initiated by lipid peroxidation, has emerged as a promising strategy for addressing drug resistance. However, the potential of targeting ferroptosis to overcome drug resistance in CS has not been systematically elucidated. Our study identifies REGγ as a critical driver of malignant progression in chondrosarcoma, with its elevated expression correlating with unfavorable patient outcomes. Loss of REGγ potentiates lipid peroxidation and modulates the activation of ferroptosis‐associated genes by enhancing WDR6 protein stability. Mechanistically, REGγ degrades WDR6 through a ubiquitin‐independent mechanism, inhibiting the ferroptosis pathway governed by the STK11/AMPK axis and consequently promoting tumor drug resistance. Additionally, RLY01, an inhibitor of the REGγ‐20S proteasome, effectively suppresses chondrosarcoma growth and sensitizes chondrosarcoma cells to cisplatin. Collectively, REGγ emerges as a highly promising therapeutic target for improving the efficacy of cisplatin and other chemotherapies in CS.

## Graphical abstract

Here, we identified REGγ as a susceptibility factor in chondrosarcoma. Our study demonstrates that abnormally activated REGγ‐20S proteasome promotes chondrosarcoma development and progression. Further validation in animal models revealed that blocking REGγ function induced ferroptosis, suppressed malignant progression of chondrosarcoma, and uncovered a novel regulatory mechanism of REGγ in chondrosarcoma. Targeting REGγ represents a promising therapeutic strategy, offering a new therapeutic target and addressing the urgent need for combination therapies in chondrosarcoma treatment.

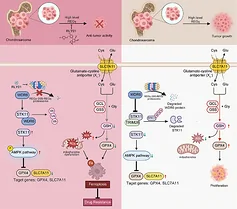

## 1 Introduction

Chondrosarcoma (CS) is a common primary malignant tumor within bone, accounting for one‐third of tumors in the skeletal system [1]. Despite significant advances in the understanding, diagnosis, and management of CS in recent years, clinical outcomes for these patients continue to be poor. As drug resistance represents a major driver of adverse outcomes in CS, effective systemic therapies remain unavailable for patients with high‐grade, unresectable, or metastatic disease [2]. Recent research indicates that IDH mutations (mIDH) serve as a defining molecular signature in chondrosarcoma, where mIDH‐positive patients demonstrate significantly reduced overall survival (OS) compared to wild‐type IDH counterparts [3]. However, the mIDH‐targeted drug Ivosidenib for CS has not demonstrated favorable long‐term safety and clinical efficacy [4]. Therefore, elucidating the drug resistance mechanisms and identifying novel therapeutic targets in chondrosarcoma is critical.

REGγ (also known as REG, PA28, or PSME), a proteasome activator belonging to the 11S proteasome activator family, selectively targets proteins for degradation in an ATP‐ and ubiquitin‐independent manner by binding to the 20S catalytic core particle [5, 6]. This noncanonical protein degradation system directly regulates cellular processes, including cell growth and proliferation, apoptosis, DNA damage response, immune response, and metabolism [7, 8, 9, 10, 11]. By influencing diverse biological pathways through protein homeostasis maintenance, REGγ plays a crucial role in tumorigenesis and metastasis [6, 12, 13]. A study indicates that inhibition of REGγ function via NIP30 enhances the sensitivity of p53‐deficient tumors to chemotherapy [14]. In this study, our proteomic analysis revealed that REGγ is closely correlated with CS prognosis and drug resistance. These results suggest that targeted inhibition of REGγ may serve as a viable strategy to overcome chemotherapy resistance in CS.

Distinct from apoptosis, necrosis, and autophagy, ferroptosis represents an iron‐dependent programmed cell death modality defined by glutathione depletion, iron overload, and lipid peroxide accumulation [15, 16, 17]. Investigating the molecular mechanisms underlying ferroptosis offers a promising therapeutic strategy for reversing drug resistance in cancer treatment. Studies report that ferroptosis demonstrates dual therapeutic advantages in multiple drug‐resistant tumor models: it suppresses primary tumor proliferation and inhibits metastatic progression, highlighting its potential for refractory bone tumor treatment [18, 19, 20]. And, activation of ferroptosis has been shown to counteract cancer cell‐acquired resistance to several anticancer agents, including lapatinib, erlotinib, and vemurafenib [21, 22, 23]. Therefore, targeting ferroptosis may represent a promising approach to overcome drug resistance and improve therapeutic outcomes in CS.

Here, we performed proteomic analysis using clinical specimens from chondrosarcoma patients. Through systematic analysis of the protein profiles, we identified REGγ as a key regulator controlling the malignancy of chondrosarcoma. Mechanistically, we established that REGγ drives tumor progression and therapeutic resistance through its specialized role as a proteasome activator, facilitating targeted protein degradation. Furthermore, our findings provide opportunities to develop effective combination therapies for CS treatment.

## 2 Results

### 2.1 REGγ Shows Upregulated Expression in CS and Correlates With Poor Prognosis

**FIGURE 1:**
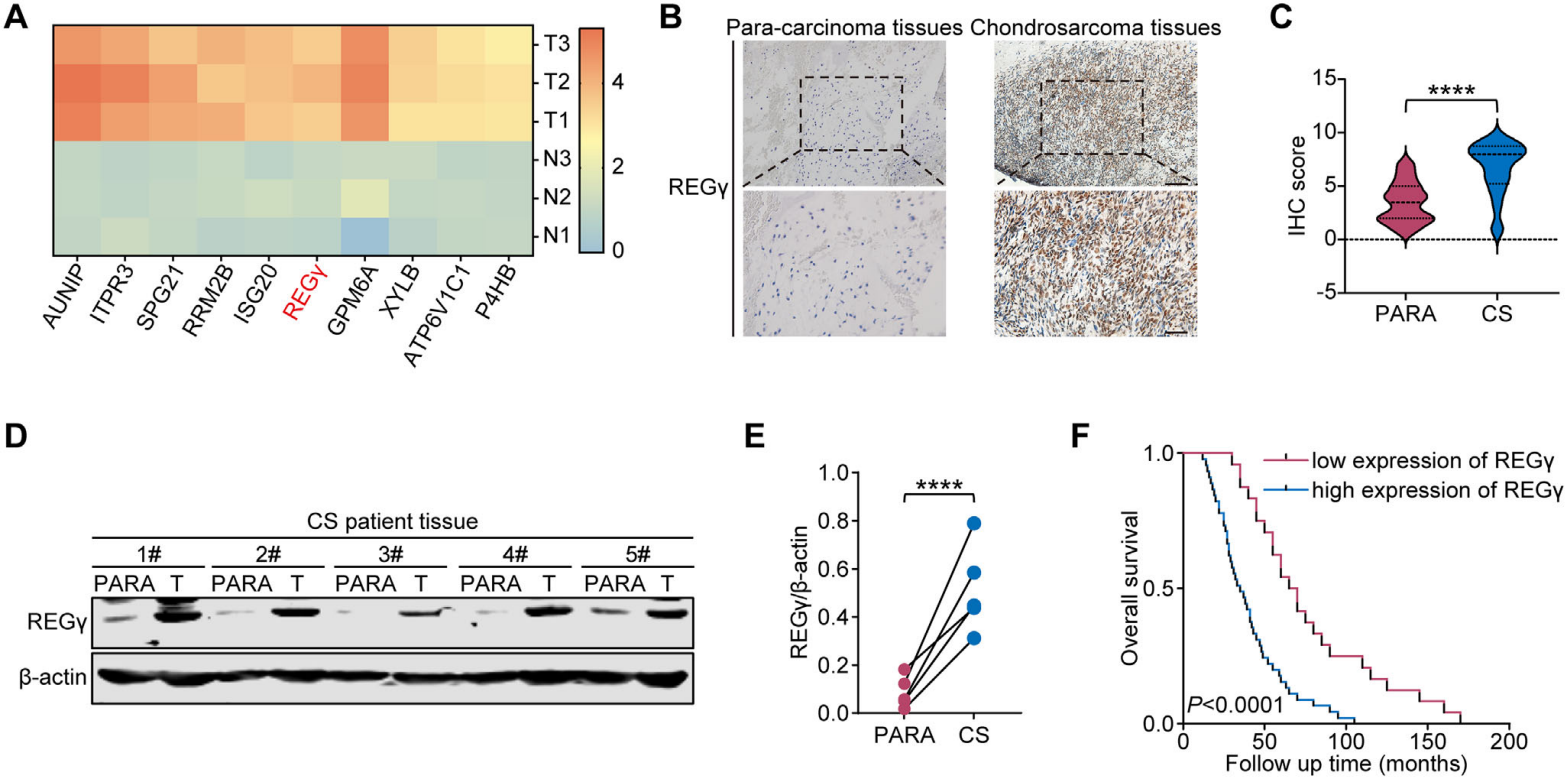
REGγ shows upregulated expression in CS and correlates with poor prognosis. (A) Heatmap showing markedly expressed proteins (*p* < 0.05) between chondrosarcoma tumors and para‐carcinoma tissues (*n* = 3). (B) Representative IHC images of REGγ expression in para‐carcinoma and chondrosarcoma tissues. Bottom: A higher magnification of sections. Scale bars: 100 µm (top), 50 µm (bottom). (C) The statistical chart showed the immunohistochemical score of REGγ. Each value represents mean ± SD (*n* = 24). (D) Immunoblotting showed that REGγ protein levels were upregulated in CS tissues. E) Analysis of REGγ protein expression in para‐carcinoma and cancer tissues from the CS patients (*n* = 5). (F) Kaplan‐Meier curve showing overall survival of CS patients with high or low REGγ expression (low expression: *n* = 24, high expression: *n* = 44). *P* values were measured by one‐way ANOVA (C, E) and a log‐rank test (F). ****p < 0.001. Data is representative of three distinct experiments and representative blots are shown from 3 independent experiments.

To comprehensively identify critical molecular drivers in CS, we performed data‐independent acquisition (DIA) proteomic profiling comparing human CS tumor tissues (*n* = 3) with matched para‐carcinoma tissue (*n* = 3). Bioinformatic analysis revealed 1456 upregulated and 1172 downregulated proteins (fold change > 1.5, *p* < 0.05, two‐sided Student's *t*‐test) in tumor specimens (Table S1). As shown in Figure 1, REGγ, a critical tumor driver, is significantly upregulated in CS tissues. To further validate its clinical relevance in CS, immunohistochemical (IHC) analysis of patient‐derived samples demonstrated elevated REGγ expression in tumor tissues (Figures 1B,C). These findings were consistently validated through Western blot (WB) and quantitative real‐time PCR (qPCR) analyses (Figure 1D,E and Figure S1A). Furthermore, survival analysis of our cohort indicated that patients with high REGγ expression exhibited shorter overall survival compared to those with low expression (Figure 1F). Collectively, these results indicate that REGγ exhibits abnormally elevated expression in CS and may serve as a predictor of poor prognosis.

### 2.2 REGγ Promotes CS Growth In Vitro and In Vivo

**FIGURE 2:**
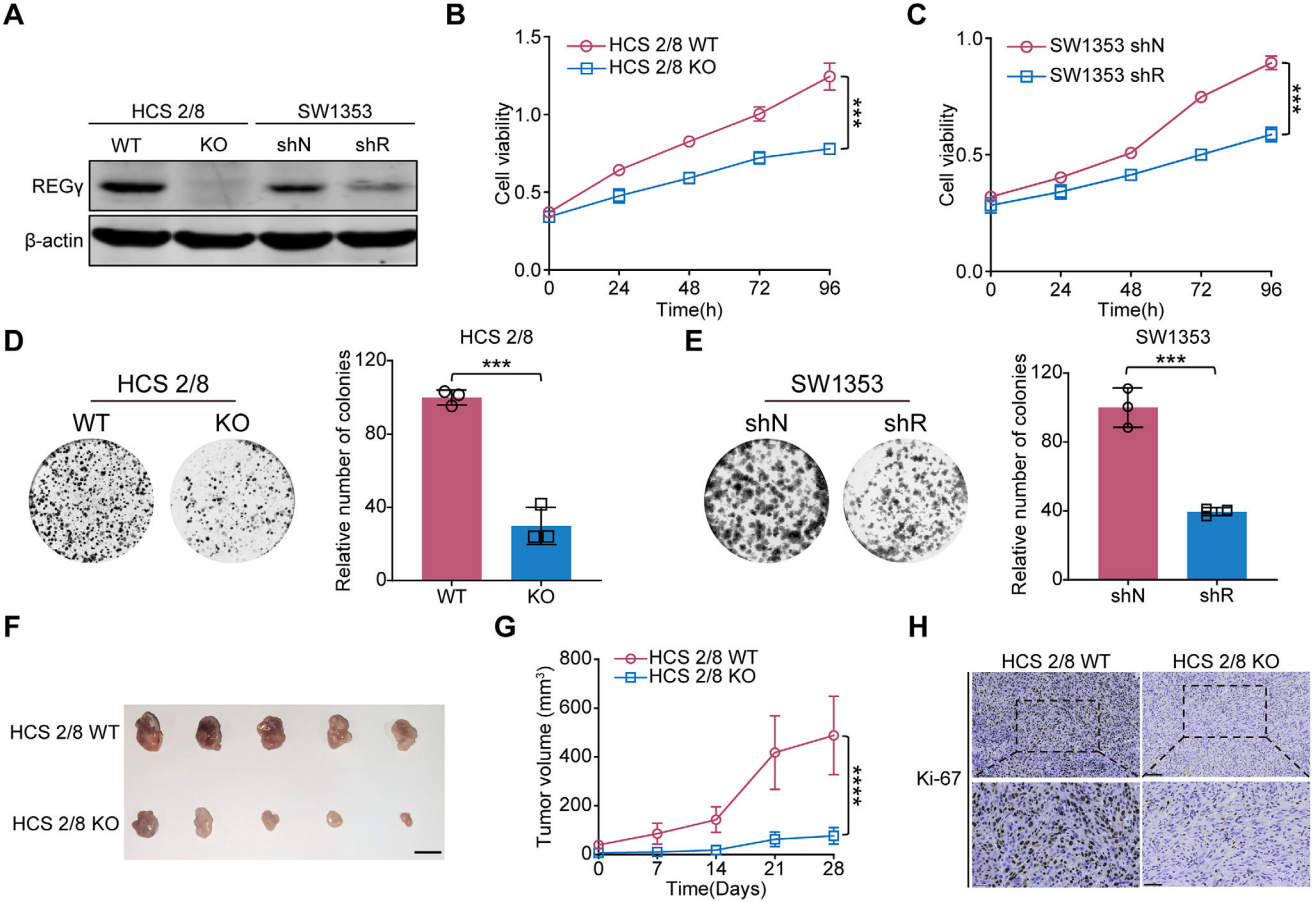
REGγ promotes CS growth in vitro and in vivo. (A) Immunoblotting showed the knockout or knockdown efficiency of REGγ in HCS 2/8 and SW13531 cells WT: wild‐type, KO: knockout, shN: with negative control shRNA, shR: with *REGγ*‐specific knockdown shRNA. (B) Relative cell viability of HCS 2/8 with or without *REGγ* knockout. Each value represents mean ± SD (*n* = 3). *P* values at point 96 h were highlighted in the figure. Statistical information pertaining to other key points can be found in the Supplemental Information. (C) Relative cell viability of SW1353 with or without *REGγ* knockdown. Each value represents mean ± SD (*n* = 3). *P* values at point 96 h were highlighted in the figure. Statistical information pertaining to other key points can be found in the Supplemental Information. (D) Colony formation assays using HCS 2/8 cells with or without *REGγ* knockout. Images of the whole plate were shown. Each value represents mean ± SD (*n* = 3). (E) Colony formation assays using SW1353 cells with or without *REGγ* knockdown. Images of the whole plate were shown. Each value represents mean ± SD (*n* = 3). (F) The picture of the tumors removed after 21 days. Scale bars: 1 cm. (G) Growth curves for HCS 2/8 xenografts with or without depletion of REGγ (*n* = 8). *p* values at point Day 28 were highlighted in the figure. (H) Representative IHC images of Ki‐67 in CS tumors. Scale bars: 100 µm (top), 50 µm(below). *P* values were measured by two‐way ANOVA (B, C, G) and one‐way ANOVA (D, E) with Tukey's multiple‐comparisons test. ^***^ *p* < 0.001, ^****^ *p* < 0.0001. Data is representative of three distinct experiments and representative blots are shown from 3 independent experiments.

To investigate the oncogenic relevance of REGγ in CS progression, we established *REGγ*‐knockout HCS 2/8 (HCS 2/8 KO) and *REGγ*‐knockdown SW1353 (SW1353 shR) cell lines (Figure 2A). Next, we explored the role of REGγ in regulating cell growth in CS. Ablation of REGγ significantly attenuated cellular proliferation, as evidenced by growth curve analysis (Figure 2B,C). Colony formation assays demonstrated that REGγ depletion robustly inhibited anchorage‐dependent growth in both HCS 2/8 and SW1353 cell lines (Figure 2D,E). Additionally, transwell migration assays and wound healing experiments revealed a marked reduction in migratory capacity upon REGγ knockdown (Figure S2A–D).

To further validate the impact of REGγ on chondrosarcoma growth in vivo, we established cell‐derived subcutaneous xenograft models and found that REGγ knockout significantly inhibited CS tumor growth (Figure 2F,G). Furthermore, IHC analysis revealed that REGγ knockout markedly reduced Ki‐67 expression, indicating inhibition of tumor cell proliferation (Figure 2H and Figure S2E). Collectively, these findings indicate that REGγ plays a critical role in promoting CS cell proliferation.

### 2.3 REGγ Mediates Ubiquitin‐Independent Degradation of WDR6

**FIGURE 3:**
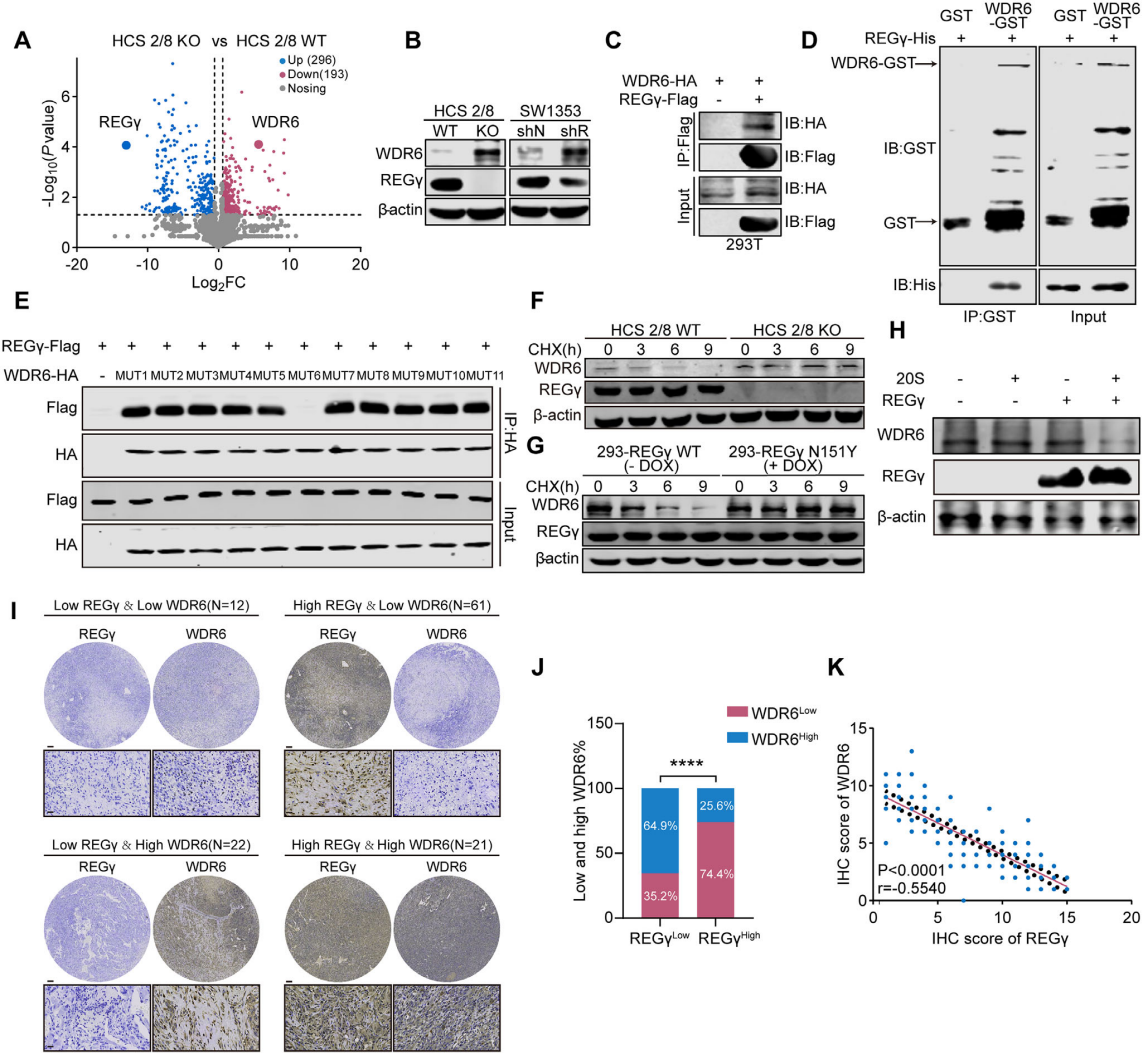
REGγ mediates ubiquitin‐independent degradation of WDR6. (A) Volcano plot showing markedly expressed proteins (*p *< 0.05, fold change <0.8 or fold change >1.5) between HCS 2/8 WT and HCS 2/8 KO cells. (B) The protein expression of WDR6 in HCS 2/8 and SW1353 cells with or without silencing of *REGγ* was detected by western blot. (C) The interaction between REGγ and WDR6 was determined by co‐immunoprecipitation and western blot analysis. (D) In vitro GST pull‐down assay detects the direct interaction between REGγ and WDR6. (E) Interaction analysis between different WDR6 mutational sites and full‐length REGγ‐Flag in HEK293T cells. (F) HCS 2/8 WT and HCS 2/8 KO cells were treated with cycloheximide (CHX, 100 µg/ml) for indicated times followed by western blot. (G) 293‐REGγ WT (active REGγ) and 293‐REGγ N151Y (inactive REGγ) cells were induced by doxycycline (DOX, 1 µg/ml) for 48 h after transfection of 4 µg Flag‐WDR6 plasmid, and then treated with CHX (100 µg/ml) for indicated times followed by western blot. (H) The in vitro ubiquitin‐independent degradation assay showed that WDR6 could be degraded by REGγ‐20S proteasome. (I) Representative IHC staining of REGγ and WDR6 expression patterns in the indicated groups are shown. Scale bar: 200 µm (top), 50 µm (bottom). (J) Statistical analysis of the correlation between REGγ and WDR6 expression patterns using Pearson 𝜒2 test in 116 CS samples. (K) Correlation between REGγ and WDR6 protein levels by IHC score. Data is representative of three distinct experiments and representative blots are shown from 3 independent experiments.

As a proteasome activator, REGγ mediates substrate degradation in a ubiquitin‐ and ATP‐independent manner, participating in multiple cellular processes including apoptosis, pyroptosis, and DNA damage [24]. To further elucidate the substrate proteins of the REGγ‐20S proteasome in chondrosarcoma, we conducted proteomic analysis and identified WDR6 as a key downstream target (Table S2 and Figure 3A). Human WDR6 is a WD40 repeat domain (WDR)‐containing protein. The WD40 repeat is a conserved structural domain composed of 40–60 amino acid residues, primarily functioning to coordinate the assembly of multiprotein complexes [25]. Using western blotting and qPCR, we validated the protein and RNA levels of WDR6 in *REGγ*‐deficient cells. Results showed that WDR6 protein levels were significantly elevated in HCS 2/8 KO and SW1353 shR cells, with no notable differences at the mRNA level (Figure 3B and Figure S3A). Furthermore, co‐immunoprecipitation and in vitro GST pull‐down assays confirmed a direct and robust protein‐protein interaction between REGγ and WDR6 (Figure 3C,D). To identify the specific domains and residues mediating the REGγ‐WDR6 interaction, we generated a series of truncated WDR6 constructs and co‐transfected them with full‐length Flag‐tagged REGγ into HEK293T cells. Co‐immunoprecipitation experiments revealed that amino acid residues 323 and 324 of WDR6 are critical for its binding to REGγ (Figure 3E and Figure S3B–D). Subsequent domain mapping analysis demonstrated that the region spanning residues 84–169 of REGγ constitutes the key structural domain responsible for interacting with WDR6 (Figure S3E). These findings suggest that REGγ regulates WDR6 expression at the protein level, indicating that WDR6 may function as a specific substrate of the REGγ‐20S proteasome.

To further validate this hypothesis, we performed cycloheximide (CHX, a protein synthesis inhibitor) chase assays, revealing that the half‐life of WDR6 was significantly prolonged in REGγ‐depletion cells compared to controls (Figure 3F and Figure S3F). Additionally, we conducted gain‐of‐function experiments using a previously established doxycycline (DOX)‐inducible 293T cell system to overexpress either wild‐type REGγ or the dominant‐negative mutant REGγ‐N151Y. Induction of WT REGγ promoted WDR6 degradation, whereas expression of the mutant REGγ‐N151Y failed to do so (Figure 3G). To determine whether REGγ directly degrades WDR6, we performed cell‐free protein degradation assays. Incubation of purified WDR6 protein with the REGγ‐20S proteasome resulted in marked degradation of WDR6 (Figure 3H). However, mutation of residues 323 and 324 in WDR6 significantly impaired the ability of the REGγ‐20S proteasome to degrade WDR6 protein (Figure S3G). Also, the REGγ N151Y‐20S proteasome showed no significant difference (Figure S3H). These results collectively demonstrate that the REGγ‐20S proteasome degrades WDR6 protein via a ubiquitin‐independent mechanism. To further investigate the link between REGγ and WDR6, we examined the expression of REGγ and WDR6 in clinical samples using IHC assay. Based on IHC scoring, CS patients were stratified into the following four groups as indicated in Figure 3I: group I (*n* = 12), low REGγ and low WDR6; group II (*n* = 61), high REGγ and low WDR6; group III (*n* = 22), low REGγ and high WDR6; and group IV (*n* = 21), high REGγ and high WDR6. As shown in Figure 3J, tumors with high REGγ expression exhibited significantly lower WDR6 staining intensity than those with low REGγ. Furthermore, we observed an inverse correlation between REGγ and WDR6 levels in clinical specimens, with high REGγ expression corresponding to low WDR6 expression (Figure 3K).

### 2.4 Loss of REGγ Promotes Ferroptosis in CS Cells

**FIGURE 4:**
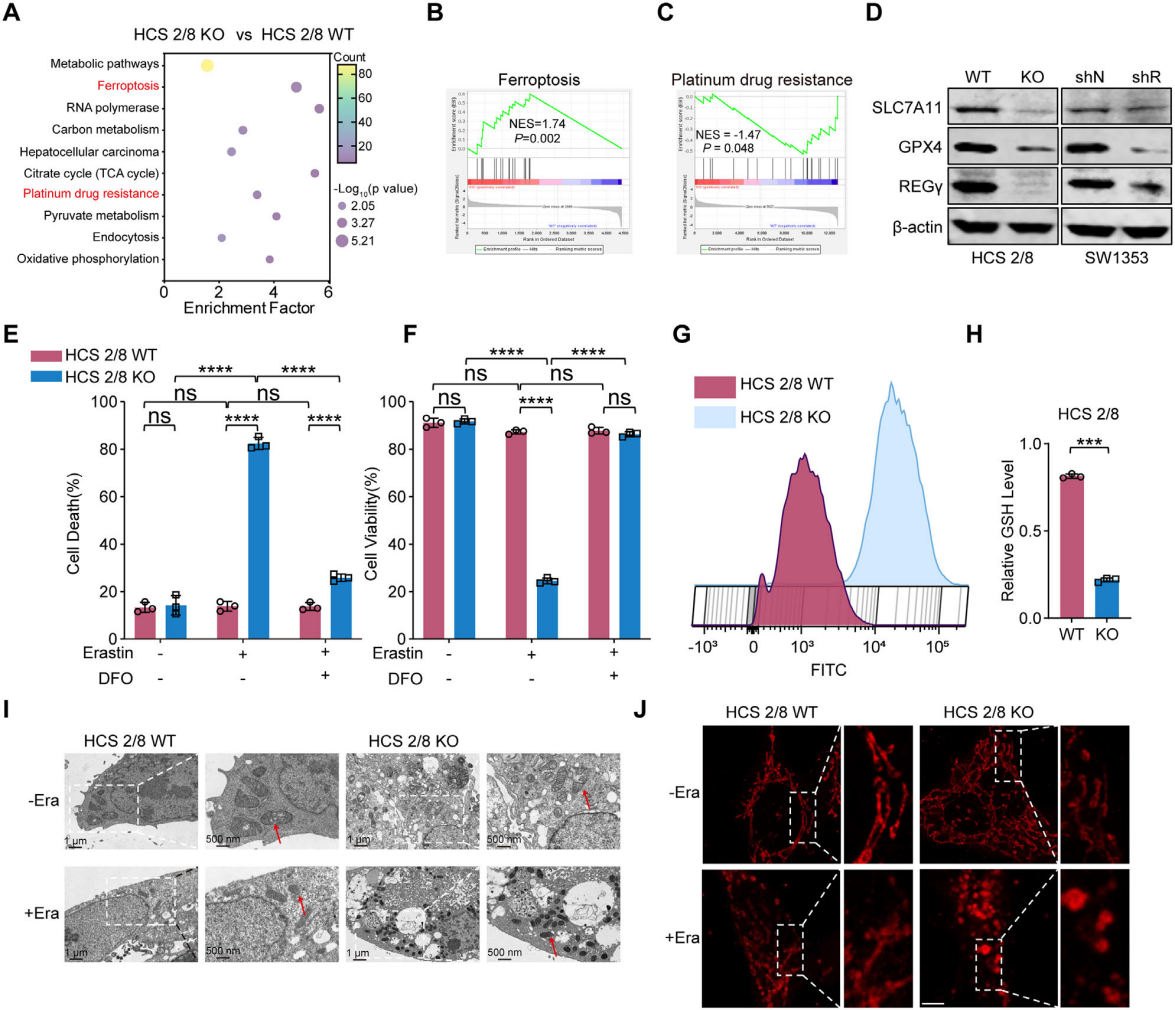
Loss of REGγ promotes ferroptosis in CS cells. (A) KEGG analysis of the differential protein between HCS 2/8 REGγ knockout (KO) and HCS 2/8 REGγ wild type (WT) cells. (B and C) GSEA was performed with ferroptosis and platinum drug resistance pathway gene set using proteomics data of HCS 2/8 cells with or without REGγ knockout. (D) GPX4 and SLC7A11 expression upon REGγ silencing as determined by western blot in HCS 2/8 and SW1353 cells. shN: with negative control shRNA, shR: with REGγ‐specific knockdown shRNA. (E) Cell death was measured after treatment with erastin (5 µM) or DFO (10 µM) in HCS 2/8 WT/KO cells (*n* = 3). (F) Bar graphs showing viability of HCS 2/8 WT/KO cells treated with erastin (5 µM) combined with DFO (10 µM) (*n* = 3). (G) Lipid peroxidation was measured by flow cytometry after C11‐BODIPY staining in HCS 2/8 WT/KO cells. (H) Bar graphs demonstrating intracellular GSH levels in HCS 2/8 WT/KO cells (*n* = 3). (I) HCS 2/8 WT/KO cells were treated with erastin (Era) and analyzed by TEM. Scale bars: 1 µm and 500 nm. The red arrows indicate the mitochondria. (J) HCS 2/8 WT/KO cells were treated with or without erastin (5 µM) and stained with COX IV, and analyzed by SIM. Scale bars: 2 µm. *p* values were measured by two‐way ANOVA (E, F) and one‐way ANOVA (H) with Tukey's multiple‐comparisons test. ^***^ *p* < 0.001, ^****^ *p* < 0.0001; ns: negative significant. Data is representative of three distinct experiments and representative blots are shown from 3 independent experiments.

To further elucidate the mechanistic role of REGγ in CS progression, we performed KEGG enrichment analysis on differentially expressed proteins, revealing significant enrichment in ferroptosis and platinum drug resistance pathways (Figure 4A). Gene Set Enrichment Analysis (GSEA) further demonstrated significant upregulation of ferroptosis‐associated pathways and downregulation of platinum‐drug resistance pathways in HCS 2/8 knockout cells (Figure 4B,C). Western blot validation confirmed that REGγ silencing markedly downregulated key ferroptosis regulators, including SLC7A11 and GPX4 (Figure 4D). These findings suggest that REGγ may modulate ferroptosis signaling to influence chondrosarcoma progression. Furthermore, REGγ deficiency significantly potentiated erastin (a ferroptosis inducer that effectively inhibits SLC7A11 activity)‐induced cell death, which was effectively rescued by deferoxamine (DFO, a ferroptosis inhibitor) (Figure 4E and Figure S4A). Stable *REGγ* knockout markedly enhanced cellular sensitivity to erastin‐mediated growth suppression, an effect reversible upon DFO treatment (Figure 4F and Figure S4B). To mechanistically confirm REGγ’s role in ferroptosis sensitization, we quantified intracellular GSH and lipid peroxidation levels. The results demonstrated that REGγ deficiency significantly promoted intracellular ROS accumulation and reduced GSH levels (Figure 4G,H and Figure S4C,D). Transmission electron microscopy (TEM) analysis further revealed that REGγ‐depleted CS cells contained shrunken mitochondria with elevated membrane density, a typical morphologic feature of ferroptosis (Figure 4I and Figure S4E). These morphological alterations were corroborated by structured illumination microscopy (SIM) using COXIV (mitochondrial marker) immunostaining (Figure 4J and Figure S4F). Collectively, these results demonstrate that REGγ regulates the ferroptosis pathway in CS, and its depletion sensitizes cells to ferroptosis.

### 2.5 REGγ Suppresses Ferroptosis by Degrading WDR6

**FIGURE 5:**
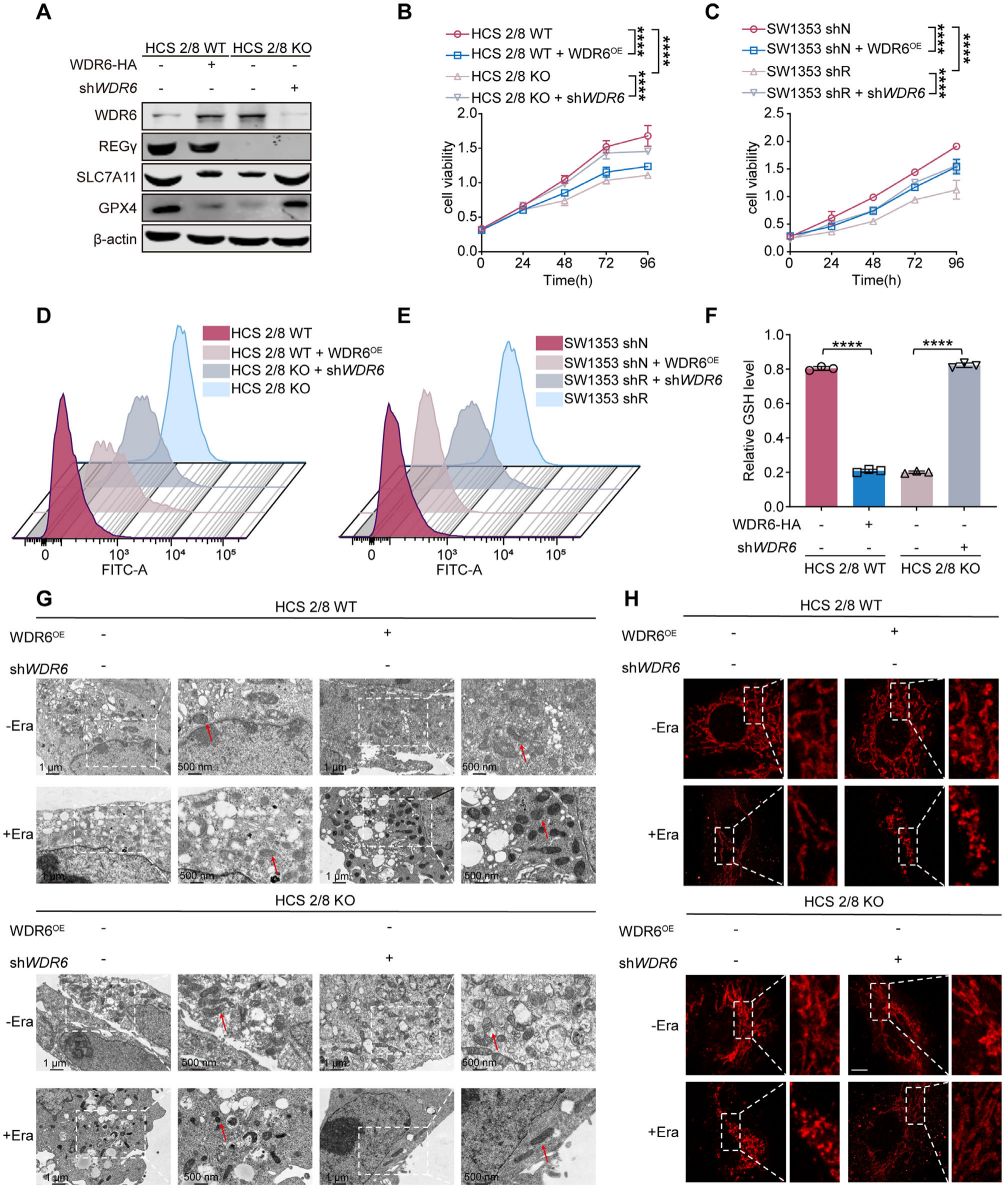
REGγ suppresses ferroptosis by degrading WDR6. (A) Immunoblotting showed the expression of GPX4 and SLC7A11 protein level with WDR6 over‐expression or knockdown in HCS 2/8 WT and HCS 2/8 KO cells. (B and C) Relative cell viability of HCS 2/8 and SW1353 with or without *REGγ* knockout and WDR6 over‐expression or knockdown. WDR6^OE^, WDR6 over‐expression. *P* values at point 96 h were highlighted in the figure. Each value represents mean ± SD (*n* = 3). Statistical information pertaining to other key points can be found in the Supplemental Information. (D and E) Lipid peroxidation was measured by flow cytometry in HCS 2/8 WT/KO cells and SW1353 shN/shR cells with or without WDR6. (F) Bar graphs demonstrating intracellular GSH levels in HCS 2/8 WT/KO cells with or without WDR6 (*n* = 3). (G) HCS 2/8 WT/KO cells with or without WDR6 were treated with erastin (Era, 5 µM) and analyzed by TEM. Scale bars: 1 µm and 500 nm. (H) HCS 2/8 WT/KO cells with or without WDR6 were treated with or without erastin (5 µM) and stained with COX IV, and analyzed by SIM. Scale bars: 2 µm. *P* values were measured by two‐way ANOVA (B, C) and one‐way ANOVA (F) with Tukey's multiple‐comparisons test. ^****^ *p* < 0.0001. Data is representative of three distinct experiments and representative blots are shown from 3 independent experiments.

Based on these findings, we hypothesize that REGγ may regulate ferroptosis by modulating WDR6 degradation. As shown in Figure 5A, the downregulation of key ferroptosis‐related proteins (SLC7A11 and GPX4) caused by REGγ deficiency could be rescued by either overexpressing or knocking down *WDR6*. Additionally, overexpression of WDR6 in HCS 2/8 WT cells and knockdown of WDR6 in HCS 2/8 KO cells led to corresponding rescue effects on CS cells' proliferation (Figure 5B,C and Figure S5A, B). These rescue effects were corroborated by measuring intracellular reactive oxygen species (ROS) accumulation and glutathione (GSH) levels (Figure 5D–F and Figure S5C). Transmission electron microscopy (TEM) and immunofluorescence (IF) further demonstrated that REGγ‐induced mitochondrial shrinkage, increased membrane density, and other hallmark features of ferroptosis were also WDR6‐dependent (Figure 5G, H and Figure S5D,E). In summary, our findings show that REGγ promotes chondrosarcoma progression through the WDR6‐dependent regulation of ferroptosis.

### 2.6 WDR6 Inhibits Ubiquitin‐Dependent Degradation of STK11 and Modulates Ferroptosis

**FIGURE 6:**
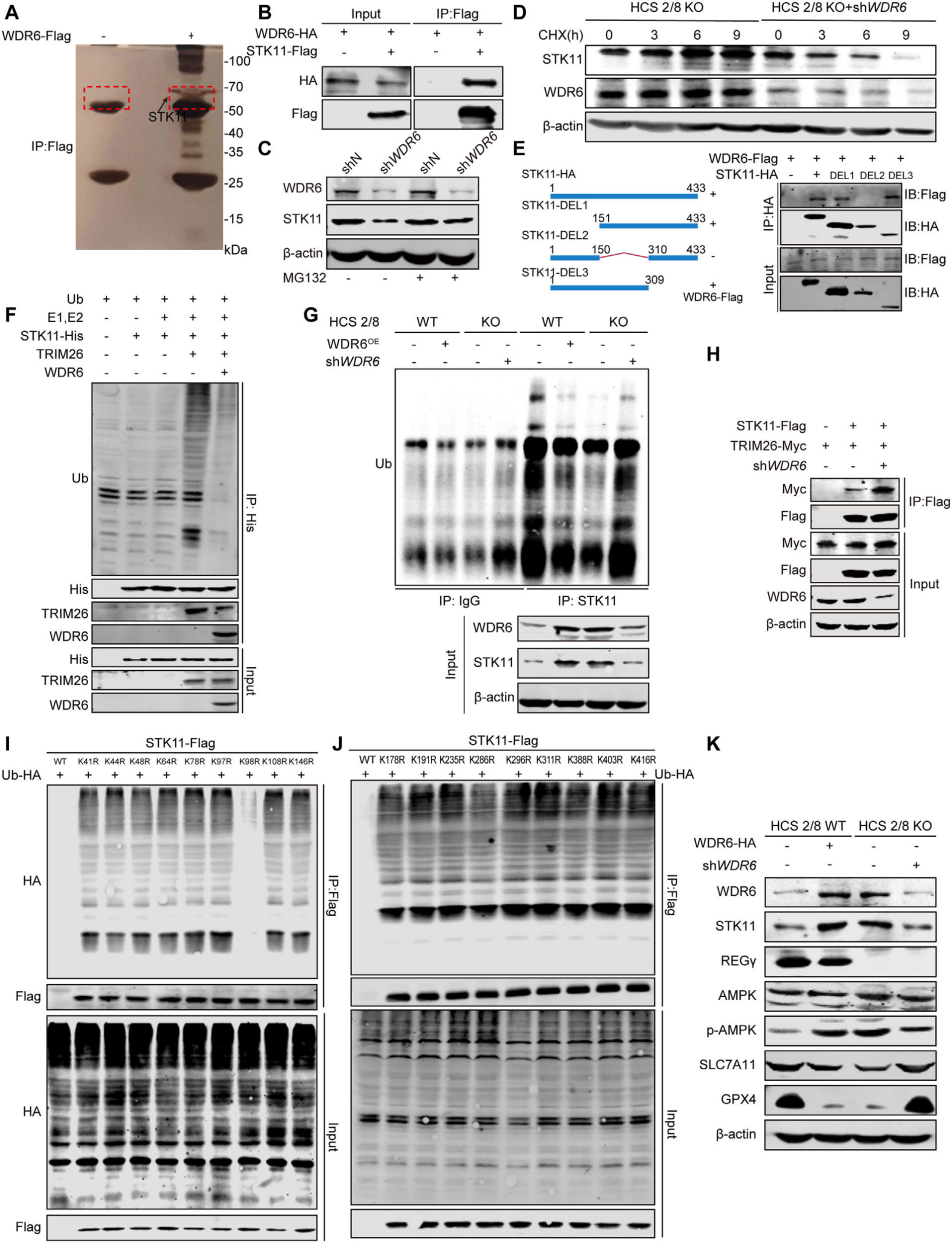
WDR6 inhibits ubiquitin‐dependent degradation of STK11 and modulates ferroptosis. (A) Silver staining of WDR6‐Flag pull‐down proteins. Whole cell lysates of HEK293T were incubated with in vitro Flag beads. WDR6‐Flag enriched bands were analyzed by MS (bands in dash lines). (B) The interaction between WDR6 and STK11 was determined by co‐immunoprecipitation and western blot analysis in HEK293T cells. (C) The protein levels of STK11 in HCS 2/8 shN/sh*WDR6* cells or control cells with or without MG132 treatment were examined using western blot. D) HCS 2/8 KO cells with or without *WDR6* stably knockdown were treated with 100 µg/mL CHX for the indicated time course. The protein levels of STK11 and WDR6 were detected by a western blot assay. (E) Immunoprecipitation assay was performed in HEK293T cells expressing Flag‐WDR6 and HA‐STK11, or HA‐STK11‐DEL1(151‐433), HA‐STK11‐DEL2(1‐150+310‐433), HA‐LKB1‐DEL3(1‐309). The HA‐tagged precipitated‐complexes were analyzed by western blot. (F) In vitro ubiquitination assay of STK11. (G) Immunoprecipitation assay was performed in WDR6 over‐expression or depleted HCS 2/8 cells for examining the ubiquitination of STK11. (H) Immunoprecipitation assay was performed in *WDR6*‐depleted or control cells to assess the interaction between STK11 and TRIM26. (I and J) Co‐immunoprecipitation assay was performed to assess the ubiquitination levels of different STK11 mutational sites in HEK293T. (K) The expression of STK11 and its downstream AMPK pathway and ferroptosis pathway were examined by western blot with or without *WDR6* knockdown in HCS 2/8 WT and HCS 2/8 KO cells. Representative blots are shown from 3 independent experiments.

Building on the above findings, we identified a novel role for WDR6 in ferroptosis regulation. However, the molecular mechanism by which WDR6 modulates the ferroptosis pathway remained unexplored. To address this, we performed immunoprecipitation coupled with mass spectrometry (IP‐MS) using WDR6 as bait, which revealed a significant interaction between WDR6 and STK11 (Figure 6A, Figure S6A, and Table S3). This interaction was further validated by co‐IP (Figure 6B and Figure S6B). In HCS 2/8 KO cells, REGγ re‐expression following WDR6 ablation failed to further suppress STK11 expression, indicating that REGγ‐mediated regulation of STK11 depends on WDR6 (Figure S6C).

To investigate the regulatory mechanism, we assessed STK11 protein levels in WDR6‐deficient CS cells treated with or without the proteasome inhibitor MG132. Results showed that MG132 treatment promoted STK11 accumulation, partially rescuing the reduction in STK11 caused by WDR6 depletion, suggesting that WDR6 regulates STK11 stability via the proteasomal degradation pathway (Figure 6C). The CHX chase assays showed a shortened STK11 half‐life in WDR6‐deficient cells (Figure 6D). Furthermore, WDR6 binds specifically to the 150–310 amino acid region of STK11 (Figure 6E). According to studies by Lei Chen et al., the E3 ubiquitin ligase TRIM26 interacts with the 243–317 amino acid region of STK11 and promotes its ubiquitination‐mediated degradation [26]. This observation prompted us to analyze E3 ubiquitin ligases enriched in IP‐MS results and those previously reported to interact with STK11, leading to the identification of TRIM26, HERC5, and HLTF. Following individual knockdown of *TRIM26*, *HERC5*, and *HLTF*, we assessed STK11 protein expression and observed that only TRIM26 silencing increased STK11 levels (Figure S6D). Interestingly, both in vitro and in vivo ubiquitination assays demonstrated that the presence of WDR6 reduced the ubiquitination level of STK11 and weakened its interaction with TRIM26. Conversely, depletion of WDR6 enhanced STK11 ubiquitination (Figure 6F,G). Co‐immunoprecipitation assays demonstrated enhanced interaction between TRIM26 and STK11 upon WDR6 depletion (Figure 6H). Furthermore, constructed three HA‐tagged truncations of WDR6. Our data indicated that the WDR6 truncation spanning amino acids 375–749 (WDR6 DELB) is the key region responsible for modulating STK11 (Figure S6E). In vitro ubiquitination assays demonstrated that the presence of WDR6‐MUTB does not affect the ubiquitination level of STK11 (Figure S6F). In addition, domain mapping assays revealed that the the 181–360 domain of TRIM26 constitutes the critical interaction domains responsible for their respective binding to STK11 (Figure S6G). Meanwhile, to identify the specific ubiquitination sites on STK11, we systematically mutated lysine (K) residues to arginine (R) within the STK11 sequence. Co‐immunoprecipitation assays demonstrated that the K98R mutation significantly reduced the ubiquitination level of STK11 (Figure 6I,J). STK11, a well‐characterized tumor suppressor, acts as a master upstream kinase that directly phosphorylates and activates the AMPK pathway [27]. Furthermore, AMPK activation has been shown to promote ferroptosis by phosphorylating BECN1, thereby inhibiting System Xc^−^ activity (an amino acid antiporter that mediates the exchange of extracellular cystine and intracellular glutamate across the plasma membrane) [28]. To elucidate the regulatory mechanism, we examined AMPK pathway activity and ferroptosis markers in the presence or absence of REGγ. The results showed that REGγ deficiency promoted WDR6 protein accumulation, thereby inhibiting ubiquitination‐dependent degradation of STK11. This enhanced AMPK phosphorylation suppressed expression of GPX4 and SLC7A11, and ultimately activated the ferroptosis pathway (Figure 6K). Furthermore, we established STK11 overexpression, knockdown, and K98R point mutation models in HCS 2/8 WT and KO cell lines. Western blot analysis revealed that the stabilization of STK11 protein led to maintained activation of the downstream AMPK pathway, leading to the downregulation of key ferroptosis‐related proteins, GPX4 and SLC7A11, which are regulated by this pathway (Figure S7A). Colony formation assays confirmed that the growth suppression induced by *REGγ* deficiency could be partially rescued by corresponding alterations in STK11 expression levels (Figure S7B). Additionally, measurements of intracellular ROS levels further validated the impact of STK11 on the ferroptosis pathway (Figure S7C). Collectively, these results demonstrate that REGγ regulates ferroptosis through the WDR6/STK11 axis, highlighting the critical role of this regulatory mechanism in CS pathogenesis.

### 2.7 REGγ Deficiency Enhances Cisplatin Sensitivity in Chondrosarcoma

**FIGURE 7:**
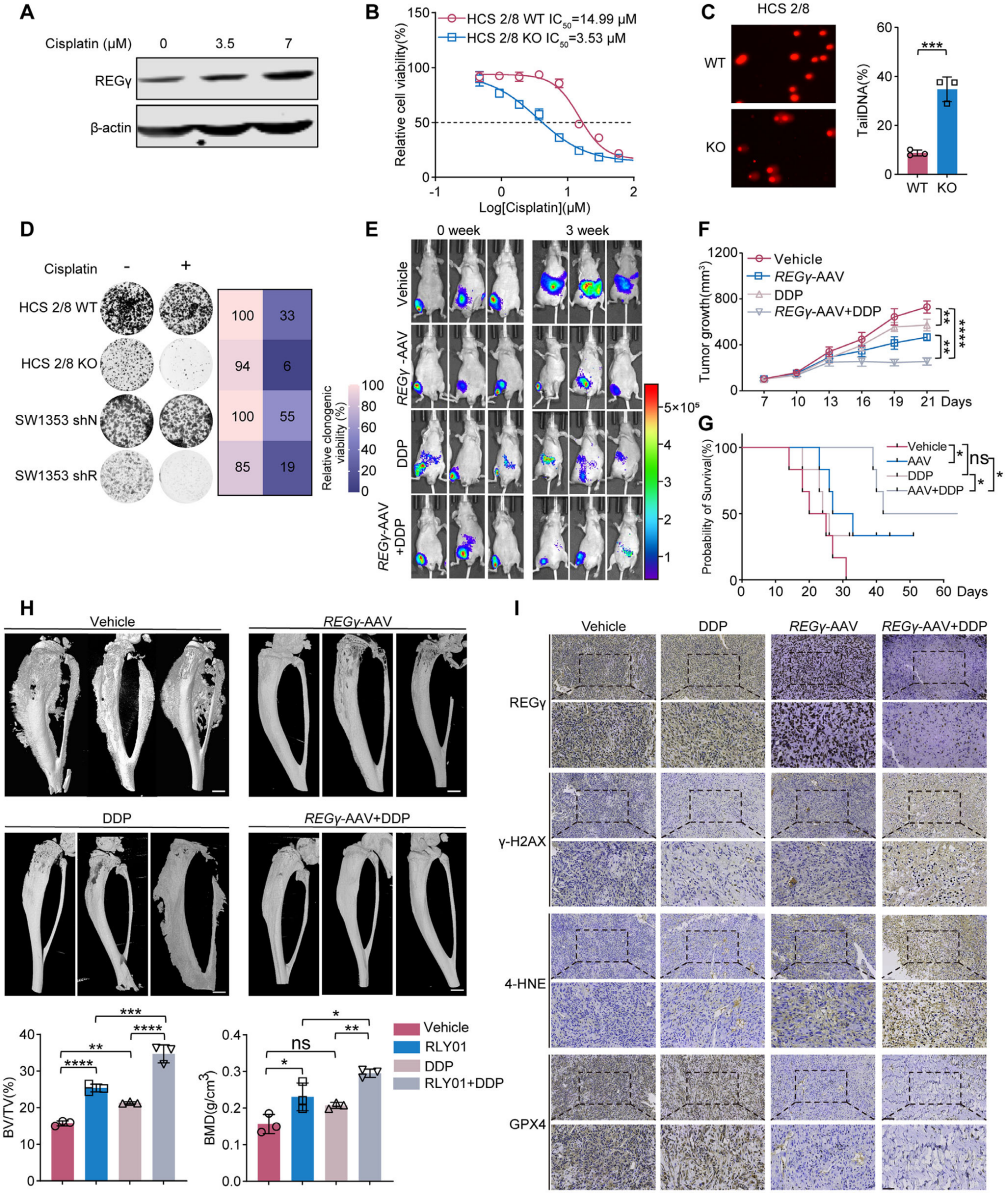
REGγ deficiency enhances cisplatin sensitivity in chondrosarcoma. (A) Immunoblotting showed the expression of REGγ in different dose of cisplatin (DDP) (0, 3.5, 7 µM) in SW1353 cells. (B) Following *REGγ* depletion in HCS 2/8 cells, alterations in the IC_50_ curve of DDP were observed. (C) The comet assay demonstrated the DNA damage conditions of HCS WT and HCS KO cells under the same cisplatin concentration (3.5 µM) treatment. (D) The colony‐formation ability of indicated HCS 2/8 and SW1353 cell lines with or without *REGγ* depletion after treatment with cisplatin (5 µM). The relative viability of cultured colonies was calculated by normalizing the HCS 2/8 or SW1353 untreated group as 100%. (E) Luminescence images of tumor bearing mice before and after treatment. (F) Growth curves for HCS 2/8 xenografts with vehicle, *REGγ*‐AAV, DDP or *REGγ*‐AAV+DDP. Values represent mean ± SD. *P* values at point Day 21 were highlighted in the figure. Statistical information pertaining to other key points can be found in the Supplemental Information. (G) Kaplan‐Meier survival curves of HCS 2/8 xenograft model mice (*n* = 6). H) Representative micro‐CT images of tibia tumor and the measurements of BV/TV and BMD in tibia. Scale bars, 1 mm. (I) Representative IHC images of REGγ, γ‐H2AX, 4‐HNE and GPX4 in resected tumors. Scale bars: 100 µm (top), 50 µm (bottom). *P* values were measured by one‐way ANOVA (C) and two‐way ANOVA (F) with Tukey's multiple‐comparisons test. Survival rate was measured by a log‐rank test(G). ^*^ *p* < 0.05, ^**^ *p* < 0.01, ^***^ *p* < 0.001, ^****^ *p* < 0.0001, ns: negative significant. Data is representative of three distinct experiments and representative blots are shown from 3 independent experiments.

GSEA analysis revealed significant enrichment of platinum drug resistance pathways following *REGγ* knockout (Figure 4C). Notably, we observed a dose‐dependent upregulation of REGγ protein levels in response to increasing cisplatin (DDP) concentrations (Figure 7A). Evaluation of cisplatin IC_50_ in chondrosarcoma cell lines HCS 2/8 and SW1353 revealed significantly lower values in REGγ‐depleted cells compared to REGγ‐WT cells (Figure 7B and Figure S8A), indicating REGγ depletion significantly sensitized CS cells to cisplatin. These findings suggest that REGγ deficiency potentiates chemosensitivity to cisplatin in chondrosarcoma, potentially overcoming drug resistance.

The deficiency of REGy leads to chromosomal instability and results in significant defects in DNA damage repair [29], therefore, we used the comet assay to demonstrate that in the absence of REGγ in CS cells, cisplatin could induce stronger DNA damage (Figure 7C and Figure S8B). And colony formation assays revealed that cisplatin exhibited the most significant inhibitory effect on CS cells under REGγ‐deficient conditions (Figure 7D). Moreover, under the same treatment conditions, the HCS 2/8 KO group showed the most pronounced apoptosis after cisplatin exposure (Figure S8C,D).

Additionally, we established a tibial orthotopic tumor implantation model of CS using HCS 2/8 cells and employed AAV adenovirus to silence *REGγ* expression in vivo. One week after model establishment, tumors were treated with cisplatin (2 mg/kg) for three weeks. Monitoring of tumor progression via IVIS live imaging and tumor size measurements revealed that REGγ downregulation significantly enhanced cisplatin sensitivity in CS tumors (Figure 7E,F and Figure S8E,F). Furthermore, *REGγ* knockdown effectively prolonged survival in cisplatin‐treated mice (Figure 7G). Micro‐CT analysis of tibial bone lesions demonstrated that the *REGγ*‐AAV+ DDP group exhibited the least bone destruction and the most preserved bone architecture (Figure 7H). Immunohistochemical analysis demonstrated that the *REGγ*‐AAV + DDP group exhibited significantly elevated expression of γ‐H2AX and 4‐HNE (a common byproduct of lipid peroxidation and serves as a well‐established biomarker for ferroptosis), along with a marked decrease in GPX4 levels, indicating that REGγ deficiency effectively enhanced the therapeutic efficacy of DDP and promoted the accumulation of ROS within tumors (Figure 7I). These findings collectively demonstrate that REGγ serves as a critical mediator of cisplatin resistance in chondrosarcoma, and its genetic ablation effectively reverses cisplatin chemoresistance in CS.

### 2.8 The REGγ‐20S Proteasome‐Specific Inhibitor RLY01 Suppresses Chondrosarcoma and Demonstrates Synergistic Efficacy With Cisplatin

In our previous research, we developed RLY01, a selective REGγ‐targeting agent, which demonstrates effective inhibition in REGγ‐high tumors [30]. In CS cells, the significant alterations in the WDR6/STK11/AMPK pathway and ferroptosis‐related proteins induced by RLY01 treatment aligned with the effects observed following *REGγ* knockout (Figure S9A). To verify the specific targeting of RLY01 to REGγ rather than REGα/β, we established *REGα/β* knockdown cell lines and treated them with RLY01 (20 µM). Western blot analysis revealed that WDR6 protein levels increased in all cell lines except those lacking REGγ, where no significant elevation was observed (Figure S9B). Furthermore, assessment of RLY01's cytotoxic effects demonstrated minimal cell death in REGγ‐deficient cells compared to other cell lines (Figure S9C). These results collectively demonstrate that RLY01 specifically targets the REGγ‐20S proteasome without affecting REGα/β function. Moreover, the inhibitory effects of RLY01 on cells were rescued by DFO (Figure S9D, E). And treatment with RLY01 led to decreased GSH levels in chondrosarcoma cells and was accompanied by accumulated ROS (Figure S9F, G). These findings demonstrate that RLY01 enhanced ferroptosis by inhibiting REGγ proteasome function, thereby suppressing CS cell proliferation and growth.

**FIGURE 8:**
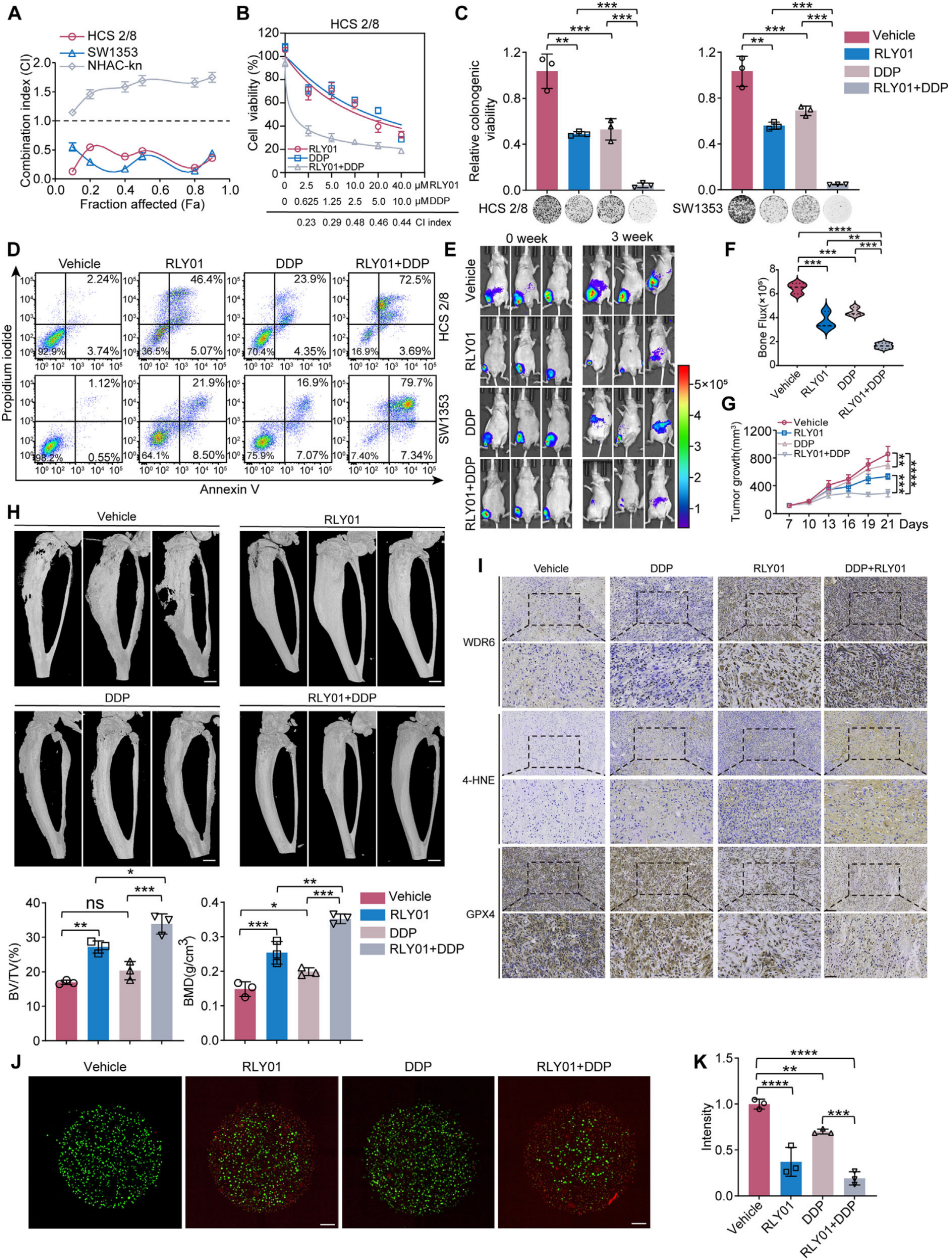
The REGγ‐20S proteasome‐specific inhibitor RLY01 suppresses chondrosarcoma and demonstrates synergistic efficacy with cisplatin. (A) Combination index (CI) for cisplatin and RLY01 co‐treatments in KNHAC‐kn (*n* = 3), SW1353 (*n* = 3) and HCS 2/8 (*n* = 3) cells. (B) HCS 2/8 cells were incubated with increasing concentrations of RLY01 and cisplatin (DDP), either alone or in combination, for 72 h, and the cell viability was determined. The CI values for the combination of RLY01 and AMG510 were calculated by using CompuSyn software. The averages and error bars represent the mean ± SD (*n* = 3). (C) Representative colony formation images of HCS 2/8 and SW1353 cells with or without RLY01 and DDP treatment. The percent cell viability is relative to the untreated controls. Each value represents mean ± SD (*n* = 3). (D) Percentage of apoptotic cells after indicated treatments in HCS 2/8 and SW1353 cells. (E and F) Luminescence images and analysis of tumor bearing mice before and after treatment. (G) Growth curves for HCS 2/8 xenografts with vehicle, RLY01, DDP or RLY01+DDP. Values represent mean ± SD. *P* values at point Day 21 were highlighted in the figure. Statistical information pertaining to other key points can be found in the Supplemental Information. (H) Representative micro‐CT images and the measurements of BV/TV and BMD of tibia tumor. Scale bars, 1 mm. (I) Representative IHC images of WDR6, 4‐HNE and GPX4 in resected tumors. Scale bars: 100 µm (top), 50 µm (bottom). (J and K) Staining images of treated organoids derived from chondrosarcoma patients. Green fluorescence represents Calcein AM staining for live cells, while red fluorescence represents propidium iodide staining for dead cells. Scale bars: 500 µm. *P* values were measured by one‐way ANOVA (C, F, H, K) and two‐way ANOVA (G) with Tukey's multiple‐comparisons test. ^*^ *p* < 0.05, ^**^ *p* < 0.01, ^***^ *p* < 0.001; ns, negative significant. Data is representative of three distinct experiments.

Notably, we revealed that REGγ promotes chemoresistance in CS by suppressing ferroptosis. While molecular therapies targeting EGFR [31], mTOR [32], and Src [33] pathways have shown promise in sensitizing CS cells to cisplatin or doxorubicin, we explored whether RLY01 could synergize with cisplatin. Using the Chou‐Talalay method to quantify drug synergy, we demonstrated strong combinatorial effects (combination index CI < 1) between RLY01 and cisplatin in CS cells (Figure 8A,B). The comet assay demonstrated that RLY01 could significantly reduce the drug resistance of chondrosarcoma and enhance the DNA damage caused by cisplatin (Figure S10A). The dual treatment significantly suppressed CS cell proliferation and enhanced apoptosis compared to monotherapy (Figure 8C,D and Figure S10B).

Furthermore, we conducted in vivo experiments to further validate the therapeutic efficacy of the combination therapy. The results demonstrated that the combination therapy group (RLY01+DDP) exhibited a robust combinatorial therapeutic effect, with chondrosarcoma tumor growth being significantly suppressed (Figure 8E–G and Figure S11A, B). Kaplan–Meier survival analysis further revealed that the combination therapy significantly prolonged the overall survival of mice (Figure S11C). Micro‐CT imaging revealed that the RLY01 and cisplatin combination significantly attenuated osteolytic damage in tibial lesions, suggesting dual efficacy in tumor suppression and bone preservation (Figure 8H). RLY01 treatment triggered a marked increase in WDR6 and lipid peroxidation (4‐HNE), coupled with suppression of the antioxidant regulator GPX4 expression, as evidenced by IHC (Figure 8I). However, gross anatomical analysis of visceral organs combined with hematoxylin and eosin (H&E) staining revealed that DDP monotherapy induced splenic toxicity (Figure S11D, E). To bridge preclinical and clinical relevance, we tested RLY01 and cisplatin in human chondrosarcoma organoids, observing synergistic growth inhibition at clinically relevant doses (Figure 8J,K). In summary, RLY01 demonstrated significant efficacy in suppressing chondrosarcoma and exhibited a marked synergistic effect with cisplatin. These findings hold promise for advancing a novel therapeutic strategy in the clinical management of chondrosarcoma.

## 3 Discussion

**FIGURE 9:**
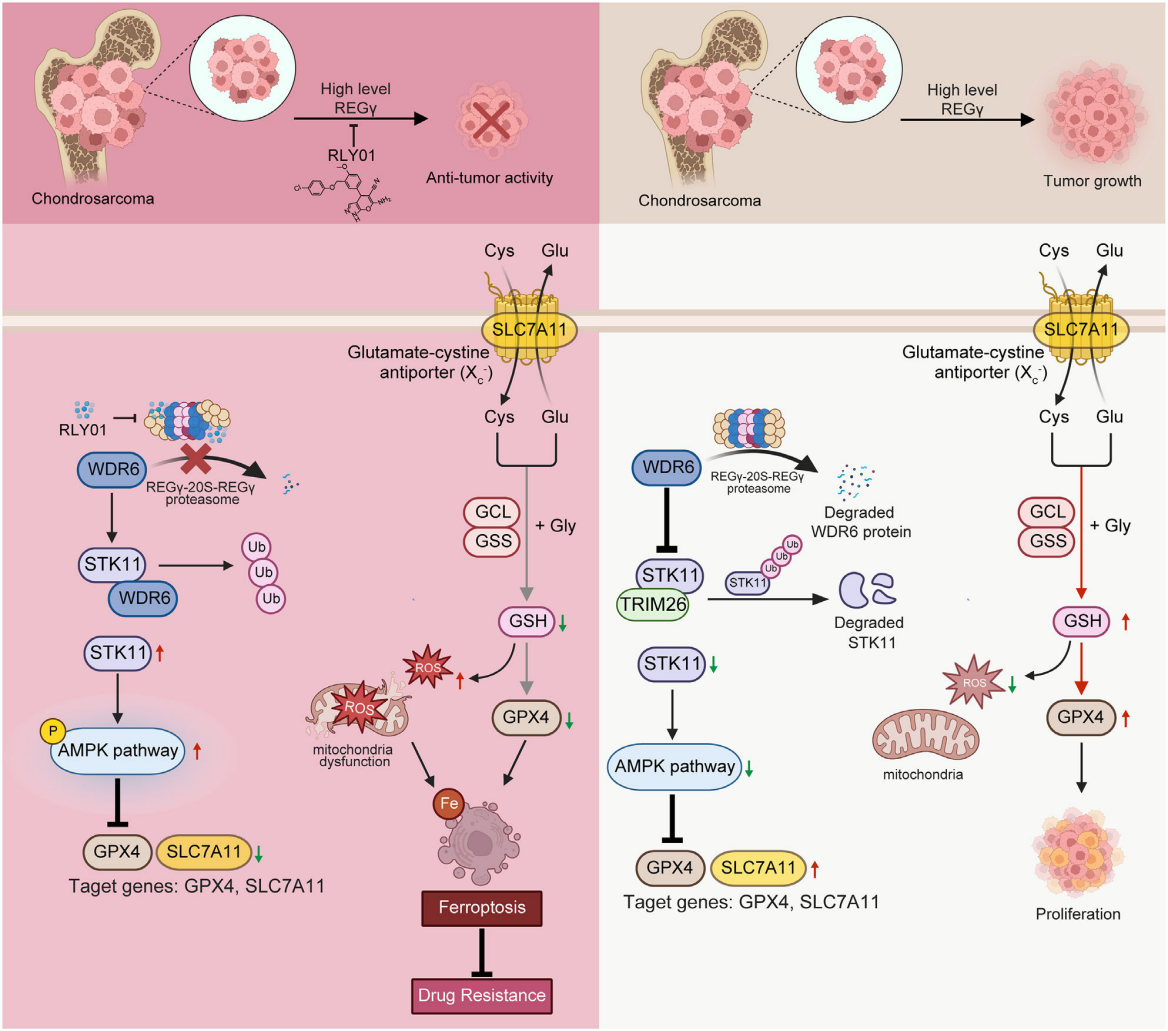
The schematic diagram shows the regulatory mechanism of REGγ in chondrosarcoma.

Chondrosarcoma (CS), a heterogeneous malignant bone tumor, accounts for approximately 20% of all bone cancers [34]. Currently, surgical resection remains the primary treatment option, while chemotherapy and radiotherapy show limited efficacy [35, 36, 37]. Over the past decade, molecular insights into CS pathogenesis have emerged, and novel therapies have entered clinical trials, yet standardized non‐surgical treatment options have yet to be established [38, 39]. Consequently, the highly heterogeneous tumor microenvironment and the development of multidrug resistance mechanisms in CS render conventional therapies struggling to overcome efficacy limitations. Here, we identified REGγ as a susceptibility factor in CS. Our study demonstrates that abnormally activated REGγ‐20S proteasome promotes CS development and progression. Further validation in animal models revealed that blocking REGγ function induced ferroptosis, suppressed malignant progression of CS, and uncovered a novel regulatory mechanism of REGγ in CS. Targeting REGγ represents a promising therapeutic strategy, offering a new therapeutic target and addressing the urgent need for combination therapies in CS treatment (Figure 9).

In this study, proteomic analysis identified REGγ as a critical vulnerability factor in CS, revealing its role as a key driver of tumor growth and survival. Both clinical samples and animal models demonstrated that blocking REGγ significantly impaired CS proliferation and survival, highlighting its potential as a promising and translatable target with functional relevance. Specifically, the REGγ‐20S proteasome drives CS progression by degrading its substrate WDR6 to regulate ferroptosis resistance. We further showed that REGγ inhibition promotes intracellular WDR6 accumulation, where WDR6 competitively binds to STK11 with the E3 ubiquitin ligase TRIM26, preventing ubiquitination‐mediated degradation of STK11. This stabilizes STK11, enhances downstream AMPK pathway signaling, and ultimately induces ferroptosis. This novel regulatory axis provides critical mechanistic insights for developing targeted therapies against CS.

Ferroptosis is a form of iron‐dependent regulated cell death, and numerous studies have demonstrated that its dysregulation frequently contributes to chemotherapy resistance and treatment failure [40, 41]. In the ferroptosis pathway, GPX4 collaborates with glutathione (GSH) to catalyze the reduction of hydrogen peroxide and organic hydroperoxides into water or corresponding alcohols, playing a crucial role in preventing ferroptosis [42]. Direct inhibition of GPX4 has been shown to induce ferroptosis. Research by Chen et al. revealed that curcumin analog‐induced androgen receptor ubiquitination suppresses GPX4, thereby triggering ferroptosis and reversing temozolomide resistance in glioblastoma [43]. Sun et al. demonstrated that ent‐kaurane diterpenoids overcome cisplatin resistance by depleting GSH to induce ferroptosis [44]. Cysteine, an essential antioxidant and rate‐limiting amino acid for glutathione biosynthesis, is regulated through the xCT system‐mediated cystine uptake [45]. Cystine is rapidly reduced to cysteine, influencing glutathione synthesis. Inhibition of the xCT system has been proven to trigger ferroptosis [46]. xCT inhibitors or genetic silencing can induce ferroptosis in head and neck cancer cells and overcome cisplatin resistance [47]. Similarly, Fu et al. found that ferroptosis induction via suppression of the Nrf2/Keap1/xCT signaling pathway sensitizes chemoresistant gastric cancer cells to treatment [48]. In the present study, we demonstrated that ablation of *REGγ* suppresses glutathione metabolism, significantly inhibiting GPX4 and xCT expression through the STK11‐AMPK pathway. This results in redox imbalance, leading to ROS accumulation that causes mitochondrial damage and triggers ferroptosis. These findings provide direct evidence that targeting REGγ enhances sensitivity to ferroptosis.

Chemoresistance in chondrosarcoma is a complex process involving multiple mechanisms. For instance, the study by Wu et al. demonstrated that cisplatin‐resistant chondrosarcoma cells exhibit highly activated glutamine metabolism via the AR/EGFR signaling pathway, which promotes NADPH production and suppresses ROS accumulation, thereby countering cisplatin cytotoxicity [49]The core of this mechanism lies in metabolic remodeling and defense against oxidative stress. Meanwhile, Chen et al. found that drug‐resistant chondrosarcoma cells increase HGF secretion, activating its receptor MET in an autocrine/paracrine manner to mediate resistance to doxorubicin [50]. This represents a classic mechanism of aberrant growth factor signaling activation, where enhanced pro‐survival signals counteract chemotherapeutic agents. In contrast, our study proposes that REGγ confers resistance by regulating the stability of specific substrate proteins WDR6 at the protein degradation level, forming a REGγ‐WDR6 axis that inhibits ferroptosis. The heterogeneity of chondrosarcoma suggests that tumor cells can utilize multiple, independent adaptive changes to resist chemotherapy. It is well established that platinum‐based drugs (e.g., cisplatin) exert therapeutic effects by inducing DNA damage, and the DNA damage repair capacity of tumor cells is a key factor in the development of resistance [51, 52]. Previous work by Li et al. directly showed that the REGγ‐proteasome can respond to drug‐induced DNA damage [53], providing a basis for REGγ’s role in the canonical DNA damage repair pathway. Our study further links REGγ to WDR6 stability and ferroptosis susceptibility, indicating that it is involved not only in direct DNA damage repair regulation but also in determining chemotherapeutic outcomes by influencing ferroptosis, thereby constructing a multi‐layered resistance barrier.

In addition to REGγ, other targets influencing chondrosarcoma resistance include AUNIP and RRM2B, both critical regulators of DNA damage repair. AUNIP physically interacts with CtIP, a requirement for CtIP accumulation at DNA double‐strand breaks [54], while RRM2B deficiency impairs p53‐mediated genomic stability [55]. Given the marked heterogeneity of CS, comprehensive elucidation of the underlying mechanisms of these proteins will expand the resistance‐associated network in CS and provide more integrated personalized therapeutic targets for future interventions. A potential limitation of our study includes the use of tumor xenograft models with two distinct CS cell lines and a single CS organoid model, which may not fully account for CS heterogeneity. Further studies employing additional patient‐derived xenograft models and CS organoid models from diverse patients may be necessary to address CS heterogeneity and reliably predict disease response to therapies.

In summary, we systematically investigated a novel mechanism by which the REGγ‐20S proteasome inhibits STK11/AMPK signaling pathway activation through degradation of its substrate WDR6, thereby modulating the ferroptosis pathway. Furthermore, the elucidation of this mechanism provides a theoretical foundation for chemoresistance in chondrosarcoma. Collectively, our study offers proof‐of‐concept evidence demonstrating that REGγ inhibition suppresses chondrosarcoma progression and enhances chemotherapy efficacy at both cellular and preclinical levels. The introduction of a novel REGγ‐20S proteasome inhibitor RLY01 in combination with cisplatin treatment provides a critical therapeutic option for future clinical development of targeted treatment strategies for chondrosarcoma.

## 4 Methods

### 4.1 Cell Lines

HEK293T, NHAC‐kn, HCS 2/8, and SW1353 cells were maintained in DMEM (Gibco, 11965092) supplemented with 10% FBS (Sunrise, SR100180.03) and 1% penicillin‐streptomycin (Aqlabtech, AQ512) in a 5% CO_2_ incubator at 37°C.

Generation of stable knockout cells. The lentiviral CRISPR/Cas9 plasmid targeting human REGγ was constructed by cloning a validated sgRNA (target sequence: CCGGCTTTCGCCGCTCACGC) into the lentiCRISPRv2 vector. HEK293T cells were co‐transfected in a 10 cm dish with a lentiviral CRISPR plasmid (4 µg), pMD2.G (1 µg), and psPAX2 (2 µg) using Lipofectamine 3000 for 48 h. The lentivirus‐containing supernatant was harvested, centrifuged at 3000 g for 10 min at 4°C, and filtered through a 0.45 µm membrane. HCS 2/8 cells were infected with the lentivirus for 24 h in the presence of 8 µg/mL polybrene, followed by puromycin selection (2 µg/mL) for 3 generations to establish stable knockout pools. Monoclonal populations were isolated by limiting dilution and validated via Sanger sequencing (T7E1 assay for indels) and western blot (anti‐REGγ antibody). Positive clones were expanded for functional studies.

Generation of stable knockdown cells. The lentiviral plasmid DNA targeting human REGγ was generated using a shRNA (Primer: GGTTCTGAGAGTAAAATTATT) in the pLKO.1 vector. HEK 293T cells were co‐transfected in a 10 cm cell culture dish with the lentiviral plasmid DNA (4 µg), pMD2.G (1 µg), and psPAX2 (2 µg) for a duration of 48 h. Next, the culture medium containing lentivirus was collected and subjected to centrifugation at 3000 g for 10 min at a temperature of 4°C. SW1353 cells were infected by virus for 24 h and further selected with puromycin for 3 generations. The selected cells were verified through western blot analysis and subsequently utilized for subsequent experiments.

### 4.2 Plasmid Construction

Plasmids used in this study are summarized in the key resource table. Plasmid of WDR6‐HA full length was used full‐length human WDR6 as the template. Target cDNA was cloned into a pcDNA3.0 (+) vector using the Hieff Clone Plus One Step Cloning Kit and corresponding primers.

### 4.3 Immunohistochemical Analysis and Hematoxylin‐Eosin Staining

Paraffin‐embedded tissues were sectioned and immunohistochemically stained for target antibodies (anti‐REGγ, anti‐WDR6, anti‐4‐HNE, anti‐GPX4, and anti‐Ki67) using the Immunohistochemistry (IHC) kit.

Tissues were embedded in paraffin and then cut into sections (4 mm thick). Immunohistochemical staining was scored according to the following standards: staining intensity (I) was classified as 0 (lack of staining), 1 (mild staining), 2 (moderate staining) or 3 (strong staining); staining percentage (P) was designated as 1 (<25%), 2 (25%–50%), 3 (51%–75%), or 4 (>75%). For each section, the semiquantitative score was calculated by multiplying I and P (which ranged from 0 to 12). Scores 0–3 was as not significant (negative), 4–8 as weakly positive and 9–12 as strongly positive. In the analysis, low expression referred to negative or weakly positive staining, while high expression indicated strongly positive staining. The log‐rank test was performed to assess statistical significance.

The target samples were fixed in 4% paraformaldehyde, dehydrated using ethanol, and subsequently embedded in paraffin for further processing. Sections approximately 4 µm thick were cut from the samples, followed by deparaffinization, rehydration, and staining with hematoxylin‐eosin (H&E). Stained slides were then evaluated using IX81 microscopy (Olympus, Tokyo, Japan).

### 4.4 Western Blotting

Cells and tissues were lysed in 1% NP‐40 buffer (50 mM Tris‐HCl [pH 7.4],150 mM NaCl, 10% glycerol, 1% NP‐40, and protease inhibitors) or RIPA buffer (50 mM Tris‐HCl [pH 7.4],150 mM NaCl, 1% Triton X‐100, 1% deoxycholate, 0.1% SDS, 10% glycerol, and protease inhibitors). The proteins were resolved on 11% SDS‐PAGE gels, and the separated proteins were subsequently transferred to PVDF membranes. The membranes were then immunoblotted with primary antibodies overnight at 4°C. Membrane incubation with Alexa‐labeled second antibodies for 1 h at 25°C, antibody‐bound proteins were analyzed by the Odyssey LICOR‐clx (USA) system. The primary antibodies used were mouse anti–β‐actin (MBL, M177‐3), rabbit anti‐REGγ (Abcam, ab157157), anti‐FLAG tag (Solarbio, K200001M), anti‐HA tag (Abcam, ab9110), anti‐WDR6 (ORIGENE, TA383311), anti‐STK11 (Proteintech, 10746‐1‐AP), AMPK (Proteintech, 10929‐2‐AP), p‐AMPK (CST, #2535T), GPX4 (Proteintech, 30388‐1‐AP), and SLC7A11 (Proteintech, 26864‐1‐AP).

### 4.5 RNA Preparation and Real‐Time (RT) PCR

Total RNA was extracted using the TRIzol reagent (Vazyme Biotech, Nanjing, China) according to the manufacturer's protocol. RNA was transcribed into cDNA using the HiScript II Reverse Transcriptase (Vazyme, Nanjing, China). Real‐time PCR was performed using the SYBR qPCR Mix (TOYOBO, China). Gene expression levels were calculated based on the 2‐ΔΔCt relative quantification methods. Each experiment was independently repeated for three times.

### 4.6 CCK‐8 Assay

Cell viability was measured using the CCK‐8 Kit (Epizyme, CX001S). In brief, cells were seeded into 96‐well plates at a density of 1500 cells/well. Cells were then cultivated at 37°C for different periods (0, 24, 48, 72, and 96 h). Upon measurement, 10 µL of the CCK‐8 mixed with 90 µL serum‐free DMEM medium was added to each well and incubated for 60 min. The supernatant's optical density (OD) values were then measured at 450 nm and compared within different groups. Each experiment was independently repeated for three times.

### 4.7 Cell Viability and Cell Death Assays

For viability assays, cells were seeded in 96‐well plates and treated with drugs for 24 h. Then, the medium with drugs was removed and replaced with fresh medium containing 10% CCK‐8 (Epizyme, CX001S). After incubation for 2 h at 37°C, the plate was measured at 450 nm.

In order to detect cell death, cells were seeded in 12‐well plates and treated with drugs for 24 h to induce cell death. Cells were digested with trypsin to obtain cell pellets, which were suspended with PBS. Cells were stained by mixing 100 µL cell suspension and 200 µL 0.02% trypan blue and incubating for 1 min, and the cells were counted with a Countess II FL automated cell counter (Thermo Fisher Scientific) to obtain the proportion of dead cells.

### 4.8 Colony Formation Assay

For the colony formation assay, cells were digested and equally seeded into 12‐well plates at a density of 1500 cells per well. The cells were then cultivated for 7 days. Afterward, the cells were fixed with 4% polyoxymethylene and stained with 0.1% crystal violet. Images of the stained colonies were captured, and the numbers of colonies was documented and compared among the different groups. Each experiment was independently repeated three times.

### 4.9 Wound‐Healing Assay

Once the cells reached approximately 90% confluence, a small area of the monolayer was disrupted by gently scratching it with a 200 µL plastic pipette tip. Subsequently, the cells were cultured for 24 h in low serum medium (1%), and images were captured using a microscope. The migration rate was calculated and compared within different groups. Each experiment was independently repeated for three times.

### 4.10 Transwell Assay

Transwell assay was carried out using transwell inserts with a 6.5‐mm diameter and 5.0 µm Pore Polycarbonate Membrane (Corning Inc., Maine, USA). Serum‐free medium containing 1.5 × 10^5^ cells complemented with different treatments to the inside compartments of the transwell inserts, whereas the lower chambers were filled with full medium containing 10% FBS. After incubation for 24 h, the lower surface of the inserts was washed twice with cold PBS, fixed with 4% polyoxymethylene and stained with 0.1% crystal violet. Each experiment was independently repeated three times.

### 4.11 Sample Preparation and Digestion

HCS 2/8 WT and HCS 2/8 KO cells were lysed by ice‐cold RIPA buffer (1% Triton, 0.1% SDS, 50 mM Tris‐HCl [pH 7.4], 150 mM NaCl, 2 mM EDTA) protease inhibitor cocktail. The mixture was centrifuged at 15 000 rpm for 10 min, and the same amount of supernatant was precipitated with methanol, chloroform and water at a ratio of 2.66:1:2. The pellets were dissolved in urea buffer containing 8 M urea and 100 mM Tris‐HCl (pH 8.5). To reduce disulfide bridges, 5 mM Tris(2‐carboxyethyl) phosphine (TCEP) was added to the solution and incubated for 20 min at room temperature. To alkylate the reduced cysteine residues, 10 mM iodoacetamide (IAA) was added to the solution and incubated in the dark at room temperature for 15 min. The urea concentration was diluted to 2 M by adding tris buffer (100 mM Tris‐HCl, pH 8.5), and 1 mM CaCl_2_ was included in the solution. The protein mixture was digested by trypsin at 37°C overnight at a ratio of 1:50 (w/w). The digested peptides were desalted with C18 Stage Tips.

### 4.12 DIA Proteomics

For DIA (Data‐Independent Acquisition) proteomic analysis, protein samples were lysed in 8 M urea buffer, reduced with 10 mM DTT, alkylated with 55 mM IAA, and digested with trypsin (1:50 w/w) at 37°C overnight. Peptides were desalted using C18 StageTips and analyzed on a Q‐Exactive HF‐X mass spectrometer coupled to a nanoLC system (gradient: 5%–35% acetonitrile over 90 min). DIA parameters included a 350–1500 m/z scan range, 25 Da variable isolation windows, and HCD fragmentation at 27% NCE. Raw data were processed using Spectronaut (v16) with a spectral library generated from parallel DDA runs or public repositories. Protein quantification was normalized to total peptide intensity, and differential analysis (*p* < 0.05, fold change > 1.5) was performed with Benjamini‐Hochberg correction. Targets were validated via Western blot or parallel reaction monitoring (PRM). QC samples were interspersed to monitor technical variability.

### 4.13 In‐Gel Digestion and MS Analysis

LC‐MS/MS–based proteomics analysis was conducted in collaboration with Shanghai Applied Protein Technology Co. Ltd. According to the methods described for the Flag‐beads pull‐down assay, Flag‐WDR6 was used to pull down target proteins from the whole‐cell lysates. SDS‐PAGE gels were stained with Silver Staining kits (Beyotime, P0017S). The entire gel was cut into pieces at the target sites (Figure 6a), which were destained, reduced, and alkylated, followed by trypsin digestion. The digested peptides were extracted, resuspended in 0.1% formic acid, and analyzed by LC‐MS/MS on a Q‐Exactive mass spectrometer (Thermo Electron). The raw MS data for each sample was combined and searched using MaxQuant 1.6.14 software for identification and quantitation analysis. The minimal scores for peptide and protein were set as 20 and 50, respectively. The FDR was set to 0.01 for analysis.

### 4.14 Co‐Immunoprecipitation

After a 48‐h transfection period with expressing plasmids, cells were harvested and lysed using a 1% NP‐40 buffer (25 mM Tris‐HCl [pH 7.4], 150 mM NaCl, 1% protease inhibitor cocktail, 1% NP‐40). After centrifugation at 15 000 g for 15 min at 4°C, the supernatant was collected, diluted 3 times with washing buffer (50 mM Tris‐HCl [pH 7.4], 150 mM NaCl, 10% glycerol and 1% protease inhibitor cocktail) and then incubated with Flag beads for 2–4 h. The immunoprecipitates were washed for 3 times by washing buffer and subjected to immunoblot analysis.

### 4.15 Drug Interaction Studies

For the analysis of the Combination index (CI), HCS 2/8 and SW1353 cells were pre‐treated with vehicle or 50 µM RLY01 for 48 h, and additionally treated with cisplatin or left untreated for 72 h. The dose of cisplatin ranged between 2.5 and 40 µM. Cell viability was measured by the CCK‐8 assay. CI was calculated using the Compusyn software version 1.0, and the synergistic effects were determined by the Chou–Talalay method [56]. A drug combination at a non‐constant ratio was used to calculate CI.

### 4.16 Flow Cytometry

For the analysis of apoptosis, HCS 2/8 and SW1353 cells were pre‐treated with 25 µM RLY01 or DMSO (vehicle) for 48 h and additionally treated with 5 µM cisplatin or double‐distilled water (vehicle) for 24 h. Subsequently, the cells were stained with the Annexin V‐APC/PI apoptosis kit (LIANKE BIOTECH) according to the manufacturer's protocol. Flow cytometry analysis was performed on a FACS Canto II flow cytometer (BD Biosciences). Each experiment was independently repeated for three times.

### 4.17 GSH Assay

The intracellular GSH level was detected using the GSH and GSSG Assay Kit (Beyotime, China). The CS cells were seeded with a density of 2 × 10^5^/well into 6‐well plates. After attachment overnight, the treated cells were lysed with freeze‐thaw cycles and centrifuged to collect the supernatant for the measurement of GSH according to the manufacturer's protocol. Absorbance was read at 412 nm using a microplate reader.

### 4.18 ROS Assay

For the measurement of ROS, cells were collected and then loaded with DCFH‐DA (Beyotime, China) in serum‐free DMEM at 37°C and incubated for 20 min according to the manufacturer's instructions. Excess DCFH‐DA was removed by washing with PBS. The ROS levels were measured through confocal fluorescence microscopy or by flow cytometry and analyzed using the FlowJo software.

### 4.19 Establishment of CDX Model

The HCS 2/8 *REGγ*‐WT and HCS 2/8 *REGγ*‐KO cells were digested and suspended in PBS, respectively, and the cell concentration was adjusted to 10^5^ cells /10 uL. 6–8 weeks old BALB/c male nude mice were housed in SPF conditions. Cells were injected into the tibial plateau of nude mice at 20 µl per spot (5 mice per group). Tumor volume was determined by calculating length × width 2 × 0.52. For anticancer drug treatment, tumor‐bearing mice were treated with individual chemotherapeutic or RLY01, as described in the figure legends.

### 4.20 In Vivo Treatments

The tumor volume of CDX model was measured every two weeks. When the tumors reached 120–150 mm^3^, treatment would begin. The treatment of usage and dosage as follows: vehicle (2% DMSO plus 5% Tween 80 plus 30% PEG‐400 in ddH2O), cisplatin (2 mg/kg, intraperitoneally, dissolved saline solution, every four days), RLY01 (20 mg/kg, intraperitoneally, dissolved in 2% DMSO plus 5% Tween 80 plus 30% PEG‐400 in ddH2O, every two days). For the chemotherapy experimental group, mice injected HCS 2/8 *REGγ* ‐WT or HCS 2/8 *REGγ‐* KO cells were randomized and treated with vehicle or cisplatin. For combination therapy, mice were randomized and treated with either vehicle, cisplatin, and RLY01 separately or cisplatin+RLY01 combination therapy for 3 weeks. Mortality and observed clinical signs will be recorded for individual animals in detail. The data were recorded, and the mice were euthanized at the satisfactory endpoint of therapy.

### 4.21 Quantification and Statistical Analysis

Experimental results were presented as mean ± SD. A two‐tailed unpaired *t*‐test was used for the comparison between two experimental groups. GraphPad Prism (Version 8.0.1) was used for statistical calculations. For comparisons of multiple groups, one‐way ANOVA was used. For multiple groups of more than one variable, two‐way ANOVA was used. A log‐rank test was performed for survival analysis.

To compare tumor volumes among different groups on a predetermined day, we initially employed Bartlett's test to assess the assumption of homogeneity of variance across all groups. When the *p*‐value of Bartlett's test was ≥ 0.05, we ran one‐way ANOVA to test overall equality of means across all groups. If the *p*‐value obtained from the one‐way ANOVA was less than 0.05, we proceeded with post hoc testing using Tukey's HSD (honest significant difference) tests for all pairwise comparisons and Dunnett's tests for comparing each treatment group with the vehicle group. If the p‐value obtained from Bartlett's test was less than 0.05, we utilized the Kruskal–Walli's test to examine the overall equality of medians among all groups. If the *p*‐value obtained from the Kruskal‐Wallis test was less than 0.05, we conducted post hoc testing using Conover's non‐parametric test with single‐step *p*‐value adjustment. This was performed for all pairwise comparisons or for comparing each treatment group with the vehicle group.

In addition, we performed pairwise comparisons without multiple‐comparison corrections and reported nominal/uncorrected *p* values directly from Welch's *t*‐test or Mann–Whitney U test. Specifically, we first used Bartlett's test to check the assumption of homogeneity of variance for a pair of groups. When the *p*‐value of Bartlett's test was ≥ 0.05, we ran Welch's t‐test. Otherwise, we ran Mann–Whitney U test to obtain nominal *P* values.

### 4.22 Study Approval

The use of human specimens in this study has been approved by the Ethics Committee of Shanghai Jiao Tong University Affiliated Sixth People's Hospital. Prior written informed consent was obtained from patients. All animal treatments were performed according to the Guide for the Care and Use of Laboratory Animals (National Academies Press, 2011). All animal procedures were approved by Shanghai Jiao Tong University Affiliated Sixth People's Hospital and were performed in accordance with IACUC guidelines.

## Author Contributions

Fanrong Liu, Shihui Shen, Dongxia Li, and Peilin Shi contributed equally to this work. FL, SS, LL, and WZ were responsible for methodology, writing, and editing. SS, JZ, LL, and WZ were responsible for conceptualization and supervision. PS and BY, ZP, WR, and LZ were responsible for software. HC, SC, and TY were responsible for the investigation. FL, SS, WS, JX, and KL were responsible for formal analysis. FL, SS, JX, LL, and ZW were responsible for validation. SS, JZ, PL, and ZW were responsible for resources. ZW, SS, LL, and WZ were responsible for funding acquisition.

## Conflicts of Interest

The authors declare no conflicts of interest.

## Supporting information




**Supporting File 1**: advs76293‐sup‐0001‐SuppMat.docx.

**Supporting File 2**: advs76293‐sup‐0002‐TableS1‐S3.xlsx.

**Supporting File 3**: advs76293‐sup‐0003‐Data.xlsx.

## Data Availability

The data that support the findings of this study are available from the corresponding author upon reasonable request.
