# Supplementary material for: REGγ Suppresses Ferroptosis and Induces Drug Resistance by Degrading WDR6 in Chondrosarcoma
Source: Adv Sci (Weinh). 2026 Jun 26:e76293. Online ahead of print. doi: 10.1002/advs.76293 (PMC13337093; doi:10.1002/advs.76293)
Supplement: Supplementary file 1 — Supporting File 1: advs76293‐sup‐0001‐SuppMat.docx. [file ADVS-9999-e76293-s002.docx]

This supplemental material contains

1.Supplemental Figure 1. REGγ shows upregulated expression in CS.

2.Supplemental Figure 2. REGγ promotes CS growth in vitro and in vivo.

3.Supplemental Figure 3. REGγ mediates ubiquitin-independent degradation of WDR6.

4.Supplemental Figure 4. Loss of REGγ promotes ferroptosis in CS cells.

5.Supplemental Figure 5. REGγ regulates ferroptosis via a WDR6-dependent way.

6.Supplemental Figure 6. WDR6 promotes STK11 protein stability to regulate ferroptosis via the AMPK pathway.

7.Supplemental Figure 7. REGγ regulates the downstream AMPK and ferroptosis pathways through STK11-mediated mechanisms.

8.Supplemental Figure 8. REGγ deficiency enhances cisplatin sensitivity in chondrosarcoma.

9.Supplemental Figure 9. RLY01 promotes ferroptosis in chondrosarcoma.

10.Supplemental Figure 10. RLY01 combined with cisplatin enhances DNA damage and cell apoptosis.

11. Supplemental Figure 11. RLY01 combined with DDP for the treatment of chondrosarcoma.

**Supplemental Figure 1.** REGγ shows upregulated expression in CS. **A**) The mRNA level of REGγ in para-carcinoma and CS tissues. *P* values were determined by two-way ANOVA with Tukey’s multiple-comparisons test. *****P* < 0.0001. Data is representative of three distinct experiments.

**Supplemental Figure 2.** REGγ promotes CS growth in vitro and in vivo. **A** and **B**) Representative images and analysis of the scratch wound-healing of HCS 2/8 and SW1353 cells with or without *REGγ* depletion. Each value represents mean ± SD (n = 3). **C** and **D**) Transwell assays using HCS 2/8 and SW1353 cells with or without *REGγ* depletion. Images of the whole plate were shown. Each value represents mean ± SD (n = 3). **E**) Representative IHC images of REGγ in resected tumors. Scale bars: 100 μm (top), 50 μm (below). *P* values were measured by one-way ANOVA with Tukey’s multiple-comparisons test. ***P* < 0.01, ****P* < 0.001. Data is representative of three distinct experiments.

**Supplemental Figure 3.** REGγ mediates ubiquitin-independent degradation of WDR6. **A**) The mRNA level of WDR6 in HCS 2/8 and SW1353 cells (n=3), Each value represents mean ± SD (n = 3). *P* values were measured by one-way ANOVA with Tukey’s multiple-comparisons test. ns: negative significant. **B** and **C**) Protein-protein interaction assays between different truncated variants of WDR6 and full-length Flag-tagged REGγ in HEK293T cells. **D**) Mapping of different WDR6 mutational sites. **E**) Protein-protein interaction assays between different truncated variants of REGγ and full-length HA-tagged WDR6 in HEK293T cells. **F**) SW1353 shN and SW1353 shR cells were treated with cycloheximide (CHX, 100 μg/ml) for the indicated times followed by western blotting. **G**) In vitro degradation assay of the WDR6-MUT6 mutant. **H**) The in vitro ubiquitin-independent degradation assay showed that WDR6 could not be degraded by REGγ (N151Y-mutant)-20S proteasome. Data is representative of three distinct experiments and representative blots are shown from 3 independent experiments.

**Supplemental Figure 4.** Loss of REGγ promotes ferroptosis in CS cells. **A**) Cell death was measured after treatment with erastin (5 μM) or DFO (10 μM) in SW1353 shN/shR cells (n=3). **B**) Bar graphs showing viability of SW1353 shN/shR cells treated with erastin (5 μM) or DFO (10 μM) (n=3). **C**) Lipid peroxidation was measured by flow cytometry after C11-BODIPY staining in SW1353 shN/shR cells. **D**) Bar graph demonstrating intracellular GSH levels in SW1353 shN/shR cells (n=3). **E**) SW1353 shN/shR cells were treated with erastin (Era) and analyzed by TEM. The red arrows indicate the mitochondria. Scale bars: 1 μm and 500 nm. **F**) SW1353 shN/shR cells were treated with or without erastin (Era) and stained with COX IV, and analyzed by SIM. Scale bars: 2 μm. *P* values were measured by two-way ANOVA (A, B) and one-way ANOVA (D) with Tukey’s multiple-comparisons test. ***P* < 0.01, ****P* <0.001, *****P* < 0.0001, ns: negative significant. Data is representative of three distinct experiments.

**Supplemental Figure 5.** REGγ regulates ferroptosis via a WDR6-dependent way. **A** and **B**) Colony formation assays using HCS 2/8 and SW1353 cells with or without *REGγ* knockout and *WDR6* over-expression or knockdown. Images of the whole plate were shown. Each value represents mean ± SD (n = 3). **C**) Bar graph demonstrating intracellular GSH levels in SW1353 shN/shR cells with or without *WDR6* knockdown (n=3). **D**) SW1353 shN/shR cells with or without *WDR6* knockdown were treated with erastin(Era, 5 μM) and analyzed by TEM. WDR6^OE^: WDR6 over expression, sh*WDR6*: *WDR6* knockdown. Scale bars: 1 μm and 500 nm. **E**) SW1353 shN/shR cells, with or without *WDR6* knockdown, were treated with erastin (5 μM), stained with COX IV, and analyzed by SIM. Scale bars: 2 μm. *P* values were measured by one-way ANOVA with Tukey’s multiple-comparisons test. ****P* < 0.001, *****P* < 0.0001. Data is representative of three distinct experiments.

**Supplemental Figure 6.** WDR6 promotes STK11 protein stability to regulate ferroptosis via the AMPK pathway. **A**) Information on the top 15 proteins identified by mass spectrometry. **B**) The interaction between WDR6 and STK11 in HEK293T cells was determined by co-immunoprecipitation and western blot analysis. **C**) Western blotting was used to detect the protein content of STK11 in cells along with the changes of REGγ and WDR6, REGγ^OE^: REGγ over expression. **D**) A western blot assay was applied to examine the level of STK11 upon depletion of distinct E3 ligases in HCS 2/8 WT. **E**) Protein-protein interaction assay between different truncated variants of WDR6 and full-length Flag-tagged STK11 in HEK293T. **F**) Western blot analysis shows the *in vitro* ubiquitination levels of STK11 in the presence of TRIM26 or WDR6-DELB. **G**) Protein-protein interaction assay between different truncated variants of TRIM26 and full-length Flag-tagged STK11 in HEK293T cells. Representative blots are shown from 3 independent experiments.

**Supplemental Figure 7.** REGγ regulates the downstream AMPK and ferroptosis pathways through STK11-mediated mechanisms. **A**) Western blot analysis demonstrates the protein expression changes in the AMPK and ferroptosis pathways following STK11 overexpression, knockdown, or K98R mutation. **B**) Colony formation assay showing changes in clonogenic ability of HCS 2/8 cells among different experimental groups. Each value represents mean ± SD (n = 3). *P* values were measured by one-way ANOVA with Tukey’s multiple-comparisons test. ****P* < 0.001, *****P* < 0.0001, ns: negative significant. **C**) Detection of intracellular ROS levels across different treatment groups in HCS 2/8 cells. Data is representative of three distinct experiments and representative blots are shown from 3 independent experiments.

**Supplemental Figure 8.** REGγ deficiency enhances cisplatin sensitivity in chondrosarcoma. **A**) Following *REGγ* silencing in SW1353 cells, alterations in the IC_50_ curve of DDP were observed. **B**) The comet assay demonstrated the DNA damage conditions of SW1353 shN and SW1353 shR cells under the same cisplatin concentration (3.5 μM) treatment. **C** and **D**) Percentage of apoptotic cells after 5 μM cisplatin (DDP) treatment in HCS 2/8 and SW1353 with or without *REGγ* depletion. **E**) The luminescence intensities of tibial tumors after treatment. **F**) The picture and the weight of the tumors removed after treatment. Scale bars: 1 cm. *P* values were measured by one-way ANOVA (B, E, F) and two-way ANOVA (C, D) with Tukey’s multiple-comparisons test. ***P* <0.01 ****P* < 0.001 *****P* < 0.0001. Data is representative of three distinct experiments.

**Supplemental Figure 9.** RLY01 promotes ferroptosis in chondrosarcoma. **A**) After treatment with RLY01 (20 μM), the changes of the WDR6, STK11, AMPK pathways and ferroptosis pathways were verified by western blotting. **B**) Western blot analysis showing the expression of the substrate protein WDR6 following RLY01 (20 μM) treatment in REGα-, REGβ-, and REGγ-deficient HCS 2/8 cell lines. **C**) Cell death was measured after treatment with RLY01(50 μM) in HCS 2/8 (n=3). Data are presented as mean ± SD. **D**) Bar graphs showing cell death in *REGγ*-depleted HCS 2/8 and SW1353 cells treated with RLY01 or DFO(n=3). **E**) Bar graphs showing cell viability of *REGγ*-depleted HCS 2/8 and SW1353 cells treated with RLY01 or DFO(n=3). **F**) Bar graph demonstrating intracellular GSH levels in *REGγ*-depleted HCS 2/8 and SW1353 cells treated with RLY01 (n=3). **G**) Lipid peroxidation was measured by flow cytometry after C11-BODIPY staining in HCS 2/8 and SW1353 cells treated with RLY01. *P* values were determined by one-way ANOVA (C, F) and two-way ANOVA ( D, E) with Tukey’s multiple-comparisons test. ****P* <0.001, *****P* < 0.0001; ns, negative significant. Data is representative of three distinct experiments and representative blots are shown from 3 independent experiments.

**Supplemental Figure 10.** RLY01 combined with cisplatin enhances DNA damage and cell apoptosis. **A**) The comet assay demonstrated the DNA damage conditions of HCS 2/8 and SW1353 cells with or without RLY01 and DDP treatment. **B**) Bar graph demonstrating the apoptosis percentage in HCS 2/8 and SW1353 cells. *P* values were determined by one-way ANOVA with Tukey’s multiple-comparisons test. ***P* < 0.01, ****P* < 0.001, *****P* < 0.0001. Data is representative of three distinct experiments.

**Supplemental Figure 11.** RLY01 combined with DDP for the treatment of chondrosarcoma. **A** and **B**) The picture and the weight of the tumors removed after treatment. Scale bars: 1 cm. **C**) Kaplan-Meier survival curves of HCS 2/8 xenograft model mice (n = 6). **D**) The gross picture of the spleen. Scale bars: 1 cm. **E**) Histological images of tissues from HCS 2/8 xenograft mouse model with or without RLY01 or DDP peritoneal injection therapy were shown. Scale bar, 100 μm. *P* values were determined by one-way ANOVA with Tukey’s multiple-comparisons test(B) and a log-rank test(C). **P* < 0.05, ****P* < 0.001, *****P* < 0.0001. Data is representative of three distinct experiments.
